# A monolithic microcavity laser with simultaneous upconversion and frequency-doubled lasing via crystal-in-glass engineering

**DOI:** 10.1038/s41377-025-02162-9

**Published:** 2026-01-26

**Authors:** Shengda Ye, Jianhao Chen, Jiayue He, Weiwei Chen, Xiongjian Huang, Xiaofeng Liu, Jianrong Qiu, Zhongmin Yang, Guoping Dong

**Affiliations:** 1 State Key Laboratory of Luminescent Materials and Devices, School of Materials Science and Engineering, South China University of Technology, Guangzhou, China; 2 School of Optoelectronic Engineering, Guangdong Polytechnic Normal University, Guangzhou, China; 3 School of Physics and Optoelectronics, South China University of Technology, Guangzhou, China; 4 School of Materials Science and Engineering, Zhejiang University, Hangzhou, China; 5 State Key Laboratory of Modern Optical Instrumentation, College of Optical Science and Engineering, Zhejiang University, Hangzhou, China

**Keywords:** Optical materials and structures, Lasers, LEDs and light sources, Integrated optics

## Abstract

This work demonstrates a novel crystal-in-glass composite structure for multifunctional micro-nano light sources in integrated photonics. Based on a Er^3+^/Yb^3+^-codoped glass-ceramic (GC) whispering gallery mode (WGM) microcavity incorporating Ba_2_TiGe_2_O_8_ (BTG) crystals, this microcavity enables dual-mode responses combining upconversion (UC) and frequency-doubled lasing. Made from a low-phonon-energy germanate glass matrix codoped with Er^3+^/Yb^3+^ for UC gain, the microcavity is crystallized to form BTG microcrystals for second harmonic generation (SHG). By leveraging the high-quality factor (Q ≈ 5.7 × 10^4^) and small mode volume, we achieve green (550 nm) and red (660 nm) UC lasing in a 30-μm-diameter microcavity with low thresholds of 13.31 μW and 12.97 μW, respectively. Benefitted from the random quasi-phase-matching (RQPM) mechanism in BTG GC, the microcavity also demonstrates an ultrabroadband frequency-doubling response from 900 to 1200 nm. By combining tapered fiber near-field coupling and femtosecond free-space pumping, we achieve simultaneous output of green/red UC lasing and frequency-doubled lasing within a single microcavity. We believe this work offers insights into hybrid material design and cooperative optical field manipulation for tunable lasers and on-chip nonlinear photonic systems.

## Abstract (web summary)

This work presents a crystal-in-glass composite doped with rare-earth ions to create a WGM microcavity that simultaneously enables efficient upconversion and broadband frequency-doubled lasing.

## Graphical abstract

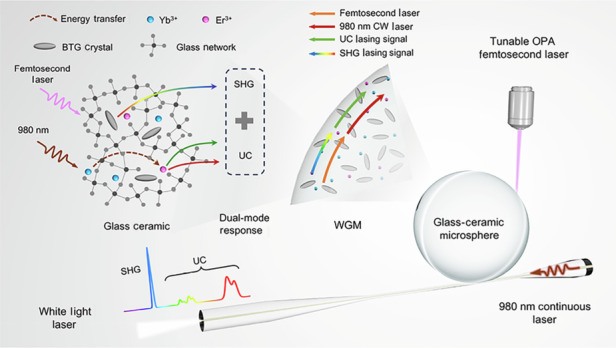

## Introduction

The rapid advancement of micro-nano photonics technology in recent years has generated a growing demand for integrated and multifunctional micro-nano light sources. Due to their ultra-high-quality factors (Q-factors) and extremely small mode volumes, whispering gallery mode (WGM) optical microcavities have emerged as a pivotal platform for enhancing light-matter interactions^1–3^. However, microcavities with a single operation mode usually cannot meet the complex demands of modern photonic devices. For example, in applications such as quantum information processing^4–10^, high-sensitivity sensing^11,12^, and optical communications^13^, there is a growing need for devices capable of simultaneous multi-wavelength laser output and nonlinear optical conversion. Consequently, the exploration of microcavity materials and structural designs capable of simultaneously supporting multiple optical processes has become a key challenge in contemporary photonics research.

While significant progress has been made in developing rare-earth-doped nanocrystal-in-glass composites for low-threshold upconversion (UC) microlasers^14,15^ and surface-crystallized glass-ceramic (GC) microcavities for efficient second harmonic generation (SHG)^16^, these demonstrations have primarily been confined to single-function optical processes. For instance, recent studies on Yb^3+^/Ho^3+^ or Yb^3+^/Er^3+^ co-doped oxyfluoride GCs incorporating NaYF_4_ nanocrystals have achieved remarkable UC lasing with ultra-low thresholds by leveraging the low-phonon-energy environment of fluoride nanocrystals^14,15^. Separately, a notable enhancement of the second-order nonlinear optical response has been demonstrated by precipitating orientation-growth nonlinear optical microcrystals and utilizing the resonant enhancement of a WGM microcavity^16^. However, these prior systems are inherently limited to either UC lasing or SHG, as the material designs are typically mutually exclusive within a single matrix, such as fluoride nanocrystals for UC or surface-precipitated nonlinear crystals for SHG.

**Fig. 1:**
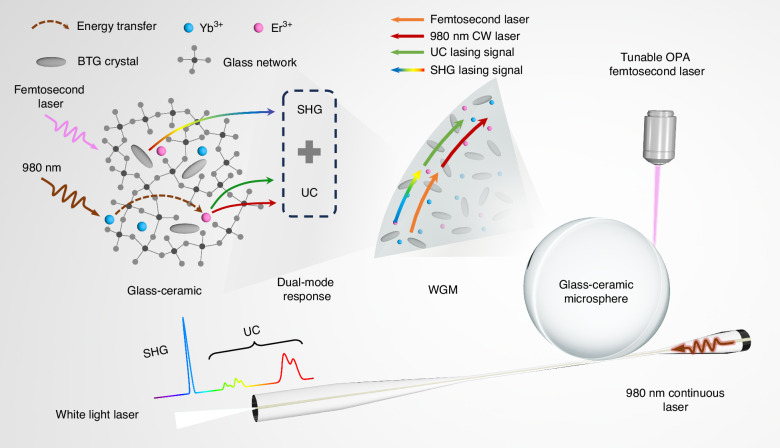
Schematic illustration of a dual-mode response microcavity laser with combined UC and SHG via crystal-in-glass engineering

To enable dual-mode synergistic responses within a single microcavity, the GC system must incorporate non-centrosymmetric crystals with strong second-order nonlinearity, while simultaneously providing efficient UC pathways for rare-earth ions. Previous studies have reported that rare-earth-ion-doped glass microcavities can achieve UC lasing^17,18^. Therefore, via crystal-in-glass engineering, we propose to develop an integrated platform within a single rare-earth-ion-doped GC microcavity, where the crystal phase supports SHG and the glass phase facilitates UC properties, enabling the simultaneous output of both UC and frequency-doubled lasing (Fig. 1).

This study focuses on a germanate GC system capable of precipitating Ba_2_TiGe_2_O_8_ (BTG) microcrystals. BTG possesses a crystal structure similar to Ba_2_TiSi_2_O_8_ (BTS), with polar TiO_5_ units aligned along the c-axis. Crucially, the incorporation of Ge^4+^, which has a larger ionic radius than Si^4+^, elongates the Ti-O bonds within these TiO_5_ units along the c-axis, enhancing spontaneous polarization. This structural modification endows BTG with superior second-order nonlinearity compared to BTS^19^. Furthermore, the germanate glass matrices generally exhibit a lower phonon energy than their silicate counterparts, which more effectively suppresses non-radiative relaxation during electronic transitions, thereby significantly enhancing the UC luminescence efficiency of rare-earth ions^20–22^. Additionally, the BTG GC exploits an intrinsic random quasi-phase matching (RQPM) mechanism. This mechanism leverages a homogeneous distribution of nonlinear crystals to randomize the wave-mixing phase, causing the harmonic output to scale with the summed intensity from individual domains and effectively circumventing the strict phase-matching requirements of conventional single crystals^23^. As demonstrated in our previous work, the RQPM mechanism enables efficient SHG over a remarkably broad bandwidth of 860-1600 nm^16^.

In this work, we have successfully fabricated Er^3+^/Yb^3+^-codoped BTG glass-ceramic WGM microcavities, allowing the first demonstration of dual-mode UC and frequency-doubled lasing. The low-phonon-energy germanate glass matrix was engineered and codoped with Er^3+^/Yb^3+^ to achieve UC gain, while the crystallization of BTG microcrystals enabled efficient SHG. By implementing a hybrid excitation scheme combining tapered fiber near-field coupling and free-space femtosecond laser pumping, we achieved simultaneous output of green/red UC lasing and visible-band SHG in a single microcavity. This work establishes a new paradigm for the development of multifunctional integrated photonic devices.

## Results

**Fig. 2:**
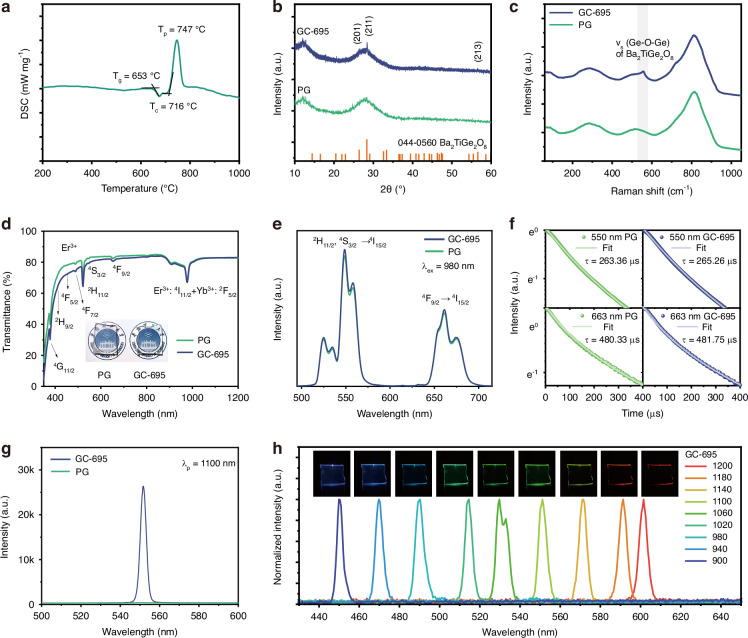
Characterization of BTG glasses. **a** DSC curve of PG sample. **b** XRD patterns, **c** Raman spectra, **d** transmittance spectra, **e** fluorescence spectra, **f** decay curves and **g** SHG spectra of the PG and GC-695 samples. **h** SHG spectra and the images of the GC-695 sample recorded with different pump wavelengths from 900 nm to 1200 nm

The precursor glass (PG) was synthesized through a melt-quenching process, which was examined first by differential scanning calorimetry (DSC) analysis (Fig. 2a). The glass transition temperature (T_g_) of the PG is 653 °C, the onset crystallization temperature (T_c_) is 716 °C, and the crystallization peak (T_p_) is 747 °C. Given the high tendency toward crystallization (ΔT = T_c_ - T_g_ = 63 °C) of the as-prepared glass, the GC were fabricated using a one-step heat treatment at temperatures slightly below T_c_ (specifically, at 685 °C, 695 °C, and 705 °C). As shown in Fig. S1 (Supporting Information), the crystallinity of the resulting GCs was found to increase with the heat-treatment temperature. We selected a heat treatment condition of 695 °C/1.5 hours for crystallization, which optimizes the optical quality of the GC microspheres, as will be discussed later. The X-ray diffraction (XRD) patterns of the PG and the GC obtained by heat treatment at 695 °C (GC-695) are shown in Fig. 2b. In the XRD curve of the PG, only amorphous humps are observed, while distinct diffraction peaks appear in the GC-695 sample. These three diffraction peaks, located at 26.5°, 28.4°, and 56.7°, correspond to the (201), (211), and (213) crystal planes of BTG (JCODS No. 00-044-0560). Additionally, the absence of any other diffraction peaks confirms the crystallization of a single BTG phase under the selected heat treatment conditions.

To further investigate the structural evolution of the glass sample during heat treatment, confocal Raman spectroscopy was employed to examine the bonding structure of the PG and GC-695 samples. As shown in Fig. 2c, the PG exhibits a typical amorphous Raman signature with three broad, featureless envelopes extending across ~200–1000 cm^−^^1^. The highest frequency envelope (700–900 cm^−^^1^) results from the overlapping of the asymmetric stretching vibration mode ν_s_ (Ti-O^*^) of apical O^*^ and the stretching vibration mode ν_s_ (Ti-O-) of apical O- in TiO_5_ units, as well as the stretching vibration mode ν_s_ (GeO_3_). The broad band centered around 520 cm^−^^1^ arises from the symmetric ν_s_ (Ge-O-Ge) stretching vibrations within the disordered glass network. A low-frequency feature near ∼290 cm^−^^1^ corresponds to localized ν(Ba-O) vibrations, with its intensity reflecting the network-modifying role of BaO^24^. After thermal treatment at 695 °C for 1.5 hours, the overall glassy background remains, but a sharp peak at 556 cm^−^^1^ emerges, corresponding to the symmetric stretching vibrations of the Ge-O-Ge bond in the ordered BTG crystal^25^. This spectral sharpening is consistent with trends observed in the controlled nucleation and crystallization of GC, where the appearance of narrower vibrational features indicates increased structural order^26,27^.

The transmittance of the samples was measured, and the results confirm that the GC obtained after heat treatment retains excellent optical transparency, with no observable turbidity in the image taken under natural light (inset, Fig. 2d). As shown in Fig. 2d, the GC-695 sample only exhibits a slightly reduction in transmittance after heat treatment, and both the PG and GC-695 samples demonstrate an average transmittance around 80% in the visible light range. Further increasing the treating temperature to 705 °C results in a significant decrease in transmittance to approximately 70% (see Fig. S2, Supporting Information), which is not conducive to the application of microcavity lasers. The doping of rare-earth ions introduces characteristic absorption peaks in the transmittance curve, more details see section S2, Supporting Information.

The UC luminescence properties of the samples were then studied. As shown in Fig. 2e, under excitation with a 980 nm laser, emission peaks at 550 nm and 660 nm were observed. Notably, the precipitation of BTG nonlinear microcrystals does not significantly affect the UC emission (more details see section S3, Supporting Information). Experimental data indicate that there is no significant change in the fluorescence lifetime of each emission band before and after heat treatment (Fig. 2f), suggesting that the heat treatment does not significantly alter the localized environment of the rare-earth ions. To further analyze the UC fluorescence mechanism of the rare-earth ions, we conducted experiments to investigate the dependence of UC fluorescence intensity on the pump power for both the PG and GC-695 samples. Experimental data reveals that under 980 nm laser pumping, the UC luminescence for both the 550 nm and 660 nm bands follows a two-photon excitation mechanism, which is in excellent agreement with the theoretical model of multi-level energy transfer from Yb^3+^ to Er^3+^ (Fig. S4, Supporting Information).

To explore the SHG properties of the Er^3+^/Yb^3+^ co-doped BTG glass system, we first study the influence of BTG crystal precipitation on SHG intensity. As shown in Fig. 2g, GC-695 exhibits a significantly enhanced SHG intensity compared to the PG sample when pumped at the wavelength of 1100 nm. Higher degree of BTG crystallinity significantly enhances the SHG intensity (Fig. S5, Supporting Information). Further investigation into the SHG response across a wide spectral range was conducted by gradually varying the pump wavelength from 900 nm to 1200 nm. The SHG response, shown in Fig. 2h, highlights the broad spectral range of the SHG effect. This is attributed to the disordered distribution of nonlinear microcrystals in the GC. The random crystal arrangement in the GC-695 sample breaks the strict phase-matching requirements typical of single-crystal materials in second-order nonlinear effects, thus promoting a broadband nonlinear response, which is one of the key advantages of GC over single-crystal materials for achieving efficient and broadband SHG^23,28^.

**Fig. 3:**
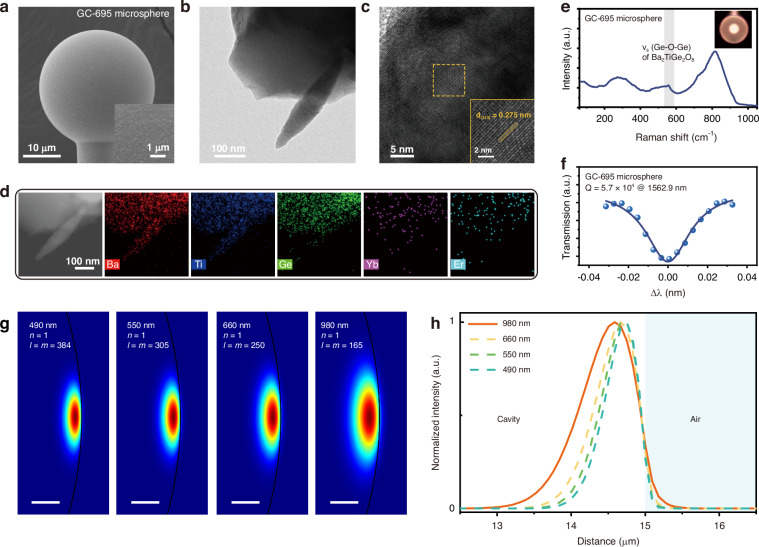
Characterizations and simulation of the GC-695 microsphere. **a** SEM images of the GC microsphere cavity, inset is the surface morphology. **b**, **c** HRTEM images of the GC-695 sample. **d** 2D element mapping for different elements of the GC-695 sample. **e** Raman spectrum and **f** Quality factor Q of the GC-695 microsphere, inset in **e** shows the micrograph of the microsphere observed with an optical microscope. **g** Mode field distribution of TE modes at wavelengths of 490 nm, 550 nm, 660 nm and 980 nm for a microcavity with a diameter of 30 μm. Scale bar: 1 μm. **h** Radial intensity distribution curves for each mode

Through structural characterizations and spectral analysis of the bulk samples, we have gained initial insights into the UC fluorescence and SHG characteristics of the Er^3+^/Yb^3+^ co-doped BTG GC. Based on this system, we prepared a series of Er^3+^/Yb^3+^ co-doped BTG GC microspheres and examined their morphology and microstructures. The micrograph of the GC-695 microsphere is shown in Fig. 3a, where good sphericity is evidenced. The inset reveals the morphology of the microcavity surface, where a scaly texture is observed. To further investigate the crystallization properties, high-resolution transmission electron microscopy (HRTEM) was performed. Figure 3b displays the low-magnification HRTEM image of the GC-695 microsphere, where needle-like substances are peeled off from the glass matrix by mechanical grinding. In the high-magnification micrograph (Fig. 3c), the lattice fringes of these needle-like microcrystals are clearly visible. The lattice spacing is measured to be 0.275 nm, which corresponds to the (310) inter-planar spacing of the BTG crystals.

To explore the element distribution in the GC-695 sample, energy dispersive spectroscopy (EDS) elemental mapping was recorded. The EDS results, shown in Fig. 3d, indicate that the Ba, Ti, and Ge elements are uniformly distributed across both the glass and crystal phases, which is consistent with their similar chemical compositions. This observation also suggests that the segregation of the elements in the BTG glass matrix does not occur during the heat treatment process.

The Raman spectrum of the GC microsphere is shown in Fig. 3e. The sharpened peak at 556 cm^−^^1^ emerges, which is attributed to the symmetric stretching vibrations of the ordered Ge-O-Ge bond in the BTG crystal, confirming again the crystallization of BTG in the microsphere, which is consistent with the results obtained from the bulk sample^25^.

In the microcavity-tapered fiber near-field coupling system, the surface roughness of the microcavity plays a crucial role in determining the coupling efficiency. To investigate the impact of heat treatment on the surface morphology, the micro-morphology of the BTG glass microsphere cavities fabricated under different heat treatment conditions (685 °C, 695 °C, and 705 °C for 1.5 hours) was analyzed. The micrographs of the PG and GC samples, shown in Figs. 3a and S6 (Supporting Information), reveal the formation of flaky-like crystals on the surface for all the Er^3+^/Yb^3+^ co-doped BTG glass microsphere cavities after heat treatment. The size of the debris continues to grow with increasing heat treatment temperature, eventually a porous network covering the rough surface is resulted. We propose that this morphology results from surface crystallization of BTG, although more rigorous experimental evidence would be help to conclusively confirm the underlying mechanism. Higher heat treatment temperatures lead to a greater degree of crystallinity, which is beneficial for increasing SHG intensity. However, excessive surface roughness, as observed in the microsphere obtained after heat treatment at 705 °C, is detrimental for subsequent near-field coupling with the tapered fiber.

To quantify the optical losses due to surface roughening, we measured the Q values of the BTG microcavities. The experimental results show that as the heat treatment temperature increases, the peak width at half height of the microcavity’s transmission peak gradually increases, as determined by the fitting with the Lorentz function. The calculated Q values for the microcavities are 8.6 × 10^4^ for the precursor, and 7.1 × 10^4^, 5.7 × 10^4^, and 3.5 × 10^4^ for GC-685, GC-695, and GC-705 samples, respectively (Figs. 3f and S7, Supporting Information). The decrease in Q values directly reflects the optical losses due to surface roughness of the microcavity. As the heat treatment temperature increases, the flaky debris on the microcavity surface grows larger and becomes more diffused, which increases scattering loss and diminishes the microcavity’s ability to confine the light field. Considering the evolution of the Q values and the importance of maintaining good crystallinity for the BTG microcrystals, we chose a heat treatment condition of 695 °C for 1.5 hours to preserve the resonant capacity of the BTG microcavities while achieving optimal optical performance.

Subsequently, we simulate the light field distributions for different operating modes within the microcavity to obtain the modal profiles, which allows us to evaluate the feasibility of achieving simultaneous UC and frequency-doubled lasing. Prior to the simulation, the refractive indices of the GC-695 sample were measured at three wavelengths of 632.8 nm, 1309 nm, and 1533 nm using the prism coupling method. The measured values were 1.83461, 1.81293, and 1.81222, respectively. Refractive indices at other wavelengths were determined by fitting with the Cauchy dispersion formula (see section S7, Supporting Information for details).

For the simulation, the finite element method was used to model a 30 μm microcavity. The fundamental modes with characteristic wavelengths of 490 nm, 550 nm, 660 nm, and 980 nm were selected to simulate the mode field distribution for both the UC (550 nm and 660 nm) and frequency-doubled lasing (490 nm), when simultaneously pumped with a CW 980 nm laser and a femtosecond pulsed laser. The results, shown in Fig. 3g, demonstrate that the mode field distributions for all four characteristic wavelengths are localized on the equatorial plane of the microcavity. As expected, the mode field area gradually increases with increasing wavelength. From the radial intensity distribution diagram (Fig. 3h), it is evident that the 980 nm WGM modes can fully cover the mode fields of both the UC laser and the SHG signal. This ensures effective energy pumping for both stimulated radiation and nonlinear conversion. Moreover, the corresponding modes for the 980 nm, 660 nm, 550 nm, and 490 nm wavelengths all form evanescent fields on the surface of the microcavity. This result indicates that, in the dual-mode operation state, both the UC and frequency-doubled lasing can be effectively generated through the near-field coupling of the tapered fiber. This simulation confirms the feasibility of achieving simultaneous UC and frequency-doubled lasing output within a single microcavity.

**Fig. 4:**
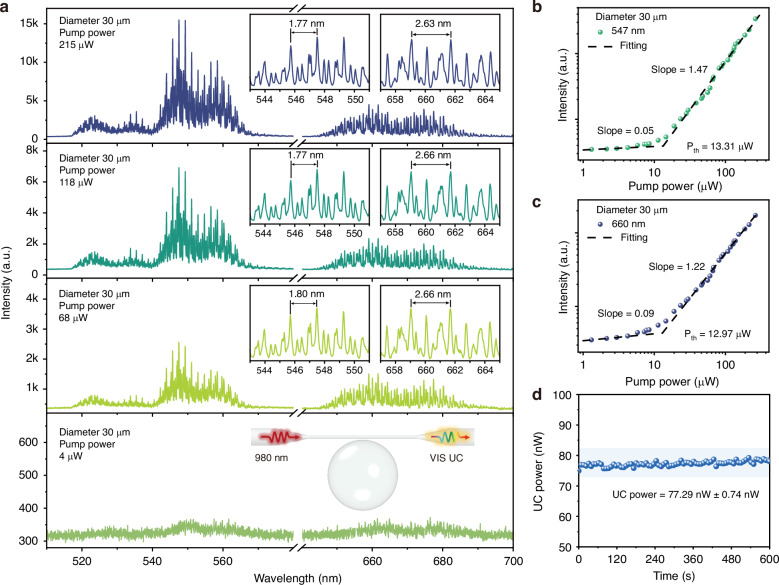
UC lasing properties of the GC-695 microcavity with a diameter of 30 μm. **a** UC laser spectra with different pumping power, insets show the zoom-in laser spectra and testing setup. **b**, **c** Power dependence curves at 547 nm and 660 nm, respectively. P_th_ refers to the laser threshold. **d** UC output stability test with output power integrated from 400 to 700 nm

The UC laser performance of the GC-695 microcavity was studied by performing measurements with a 30 μm GC-695 microcavity, which was pumped by a 980 nm CW laser coupled with a tapered fiber. As shown in Fig. 4a, when the pump power is set to 68 μW, typical WGM periodic comb signals are generated in both the green and red light bands. This indicates that UC lasing from the microcavity is achieved, with the respective stimulated radiations corresponding to the Er^3+^ transitions: ^2^H_11/2_, ^4^S_3/2_ → ^4^I_15/2_ (green) and ^4^F_9/2_ → ^4^I_15/2_ (red). As the pump power increases, both the green and red resonant peaks remain stable. The measured free space range (FSR) for the 30 μm microcavity at the 547 nm and 660 nm bands is approximately 1.77 nm and 2.63 nm, respectively, which are consistent with the theoretical values (section S7, Supporting Information). More details are revealed by the high-resolution lasing spectra. The multiple laser peaks within a single FSR indicate the presence of higher-order modes, which result from the sufficient gain regions located at positions away from the surface of the BTG GC microcavity^15^.

The UC microcavity laser intensity as a function of pump power was recorded and plotted in logarithmic coordinates (Fig. 4b, c). A distinct inflection point appears on the power-dependent curve, indicating the onset of lasing with relatively low thresholds of 13.31 μW at 547 nm and 12.97 μW at 660 nm (A more detailed comparison with other materials is shown in Table S4, Supporting Information). These values confirm the ability to achieve UC lasing with a relatively low pump power in the GC-695 microcavity. The stability of the UC laser output power from the microcavity was tested. The results (Fig. 4d) show that the system exhibits excellent stability, with a relative standard deviation of the output power within 600 s being 0.96%.

To investigate the influence of the size effect on the UC laser performance, GC microcavities with a larger diameter of approximately 45 μm were fabricated under heat treatment at 695 °C for 1.5 hours. As shown in Fig. S10a (Supporting Information), the larger microcavity exhibits the same UC laser evolution process as the 30 μm microcavity when the pump power increases. However, increasing the diameter of the microcavity leads to a reduction in the mode spacing of the laser, resulting in denser, comb-like spectra. The measured FSR for the 45 μm microcavity in the 547 nm and 660 nm bands is found to be 1.19 nm and 1.78 nm, respectively. These values are highly consistent with the theoretical prediction that the FSR is inversely proportional to the microcavity diameter (FSR ∝ 1/D, Section S7, Supporting Information).

The UC laser intensity as a function of pump power for the microcavity with 45 μm diameter is also recorded and plotted (Fig. S10b and S10c, Supporting Information). The laser thresholds in the 547 nm and 660 nm bands are found to be 18.47 μW and 14.76 μW, respectively. This result shows that as the microcavity diameter increases, the laser thresholds slightly increase, likely due to the larger cavity volume, which may result in a lower local energy density. The experimental data reveal that smaller-sized microcavities tend to exhibit laser spectra with more separated modes and lower lasing thresholds, making them more suitable for UC microcavity lasers.

**Fig. 5:**
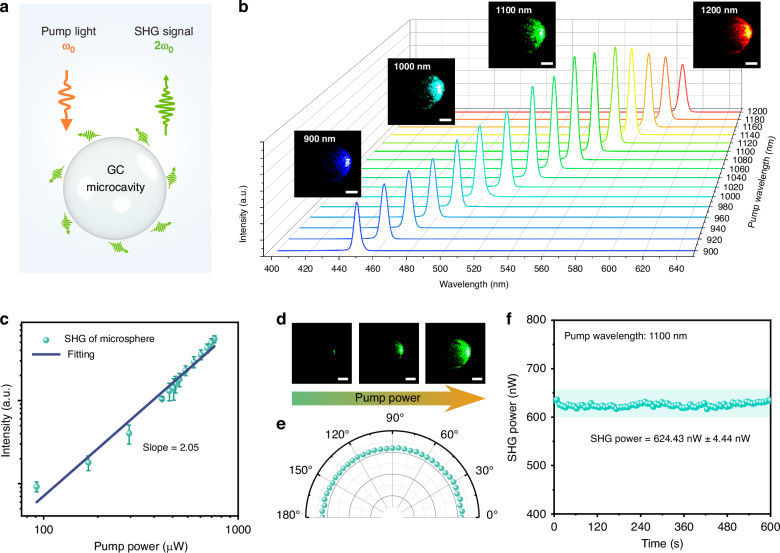
Experimental setup and SHG in the GC-695 microcavity. **a** Schematic diagram of femtosecond pulse free-space pumping. **b** SHG spectra of the GC-695 microcavity recorded at pumping wavelengths from 900 nm to 1200 nm at a constant pumping power of 500 μW, insets are the corresponding micrographs at different pumping wavelengths, scale bar: 10 μm. **c** Power dependent curve with error bar showing the standard deviation, **d** micrographs of the microcavity taken with increasing pump power (scale bar: 10 μm), and **e** polar plot of the SHG intensity for the GC-695 microcavity as a function of pump polarization at the wavelength of 1100 nm. **f** SHG output stability test

To verify the nonlinear optical properties of the GC-695 microcavity, an experimental setup utilizing femtosecond pulse free-space optical pumping with a confocal microscope system was designed, as illustrated in Fig. 5a (more details are shown in characterizations section). Firstly, a pump wavelength-dependent measurement was conducted. In this experiment, the femtosecond pulse pump power was fixed at 500 μW, and the wavelength ranged from 900 nm to 1200 nm with an interval of 20 nm. The GC-695 microcavity was successively pumped across this wavelength range. As shown in Fig. 5b, within the specified pump wavelength range, the GC-695 microcavity successfully achieved SHG covering the 450–600 nm visible spectral region. As the pump wavelength increases, the intensity of the SHG signal grows first and then drops. The SHG signal intensity reached its peak within the wavelength range of 1080 nm to 1120 nm.

Then, the SHG properties were further studied with the pump wavelength of 1100 nm. The pump power dependence of the SHG intensity was recorded, as shown in Fig. 5c. The SHG intensity exhibits a clear quadratic relationship with the pump power, confirming the dominance of second-order nonlinear characteristics in the BTG GC system. The SHG conversion efficiency of the GC-695 microcavity was tested to be 2.7 × 10^−^^4^ @ 1100 nm, which is close to the surface crystallized BTS GC microcavity^16^. Figure 5d presents the corresponding micrographs of the GC-695 microcavity under pumping with increasing pump powers. The laser damage threshold of BTG GC is 68.98 × 10^−^^2^ mW μm^−^^2^ @ 1100 nm. The pump polarization dependence of the SHG intensity was also measured for the GC-695 microcavity. As shown in Fig. 5e, as the polarization direction of the pump light rotates from 0° to 180°, the polar plot of the SHG signal intensity exhibits a hemispherical profile. This result indicates that the SHG response in the BTG GC microcavity does not exhibit polarization dependence, which is consistent with the results of nonlinear microcrystal-doped glass composite material^29^. This behavior is attributed to the complete randomization of the spatial orientation of BTG microcrystals within the microcavity. The random distribution of the microcrystals effectively eliminates the intrinsic polarization selection rules that typically dominating in single-crystalline materials. As a result, the BTG GC microcavity exhibits an isotropic nonlinear optical response, making it highly suitable for broadband applications without the need for specific polarization alignment^16^. The stability of the SHG output power of the microcavity was tested. The results (Fig. 5f) indicate that the system exhibits excellent stability, with a relative standard deviation of 0.71% within 600 s.

Finally, we employed a dual-pumping scheme using a tapered fiber and a confocal microscope system to couple the 980 nm CW laser and a tunable femtosecond pulsed laser into the microcavity. The pump coupling configuration is illustrated in Fig. 1. Through this setup, dual-mode cooperative output combining UC and frequency-doubled lasing was successfully collected via the same tapered fiber. During the pump-coupling process, precise alignment of the microcavity relative to the tapered fiber was achieved using a three-dimensional nanopositioning stage. It is worth emphasizing that the experimental procedure has been systematically repeated across multiple microcavity samples and coupling cycles, confirming the reproducibility of the dual-mode lasing output under consistent alignment conditions. Notably, the femtosecond pulses were pumped at the edge of the microcavity. By finely adjusting the coupling position, the leakage region of the SHG signal was made to spatially overlap with the upconversion resonant ring produced under 980 nm CW pumping. This spatially cooperative interaction of multi-wavelength modes enables the tapered fiber to simultaneously collect both UC and frequency-doubled signals from the same microcavity, demonstrating a robust and repeatable route for integrated dual-mode operation.

**Fig. 6:**
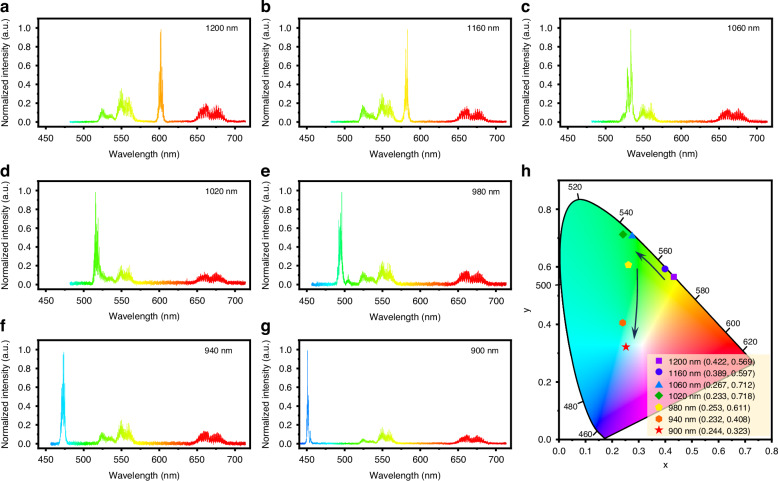
The laser spectra and CIE coordinates of the dual-mode laser. The dual-mode output spectra of the GC microsphere with different femtosecond pulse pump wavelengths of **a** 1200 nm, **b** 1160 nm, **c** 1060 nm, **d** 1020 nm, **e** 980 nm, **f** 940 nm and **g** 900 nm. **h** Tunable CIE coordinates of the dual-mode microcavity output

As shown in Fig. 6a–g, with the 980 nm CW laser pumping and the femtosecond pulse wavelength adjusted between 900 nm and 1200 nm, distinct features of the dual-mode output spectra of the microcavity were observed. Notably, periodic comb-like UC laser spectra are detected at wavelengths of 550 nm and 660 nm, confirming that the microcavity successfully maintains the upconversion lasing process of Er^3+^.

Additionally, a series of narrowband SHG signal peaks was observed at half the femtosecond pump wavelengths. Notably, the SHG signal output via the tapered fiber coupling method also demonstrates ultra-broadband response characteristics across the 900–1200 nm range. Combined with the multi-wavelength resonance features of the WGMs, it enables the microcavity output to cover the 450–600 nm visible spectral region. Furthermore, benefited from the near-field coupling method, the splitting mode of the SHG signal output from the tapered fiber is sharper, thereby better preserving the resonance characteristics of the microcavity.

Figure 6h presents the CIE coordinates of the dual-mode signals output as the femtosecond pump wavelength varies from 900 nm to 1200 nm. By tuning the pump wavelength, the dual-mode signals produce a wide range of CIE coordinates. Specifically, when the femtosecond pump wavelength is set to 900 nm, the output signals yield CIE coordinates near white light (0.33, 0.33), with values around (0.244, 0.322). By adjusting the intensity ratio between the SHG and UC lasing signals, the CIE coordinates of the output signal can be further fine-tuned, as demonstrated in Figs. S13 and S14 (Supporting Information). The tunable SHG signal not only amplifies the intensity in the upconversion regions but also fills the gap formed by the non-emitting regions of the Er^3+^ UC laser (Fig. S13, Supporting Information). The cooperative operation of the dual-mode signals significantly enhances the output spectral tunability of the microcavity. Especially, the demonstration of near-white light lasing underscores its immense potential in the development of tunable multi-wavelength micro-nano photonics technology^30,31^.

## Discussion

In this work, a crystal-in-glass composite architecture combined with a rare-earth ion doping strategy has been developed to realize a multifunctional WGM microcavity capable of simultaneous high-performance UC lasing and broadband response SHG. Based on a 30 μm diameter GC microcavity incorporating Er^3+^/Yb^3+^ co-doped Ba_2_TiGe_2_O_8_ microcrystals, green and red lasers and visible-band SHG are simultaneously achieved, significantly reducing the complexity of multifunctional integration. Crucially, the GC material system is highly compatible with planar waveguide architectures, suggesting a viable pathway toward scalable on-chip integration. Our work offers a novel approach toward high-performance integrated photonic chips with multi-wavelength cooperative operation, showing promising potential for applications in fields including tunable multi-wavelength lasers and on-chip nonlinear light sources.

## Materials and methods

### Fabrication of the bulk glass

The glass precursor with a nominal composition of 40BaO-20TiO_2_-40GeO_2_-0.5YbF_3_-0.1ErF_3_ (mol%) were prepared from analytical-grade raw materials (BaCO_3_, TiO_2_, GeO_2_, YbF_3_, ErF_3_). In a typical batch, precisely weighed powders of 30 g were homogenized by grinding in an agate mortar, and then melted in a covered platinum-rhodium crucible at 1350 °C for 30 minutes under ambient atmosphere. Prior to casting, the furnace temperature was reduced to 1300 °C, whereupon the molten glass was rapidly poured onto a preheated copper plate and immediately pressed with a second copper plate to facilitate quenching into a transparent glass sheet. To relieve internal stress, the as-quenched glass sheets were promptly transferred to a preheated muffle furnace for annealing at 630 °C for 1 hour followed by controlled cooling to room temperature over 10 hours. Annealed samples were then cut and polished. Crystallization was subsequently induced by the heat treatment of the glass precursors in a muffle furnace at different temperatures for 1.5 hours to precipitate BTG microcrystals.

### Microsphere fabrication

Microsphere fabrication involved melting of the precursor glass, fiber drawing, microsphere formation, and crystallization processes. Using the identical glass composition of 40BaO-20TiO_2_-40GeO_2_ (mol%) and the melting protocol established for bulk samples, the glass melt was prepared. Afterwards, the furnace temperature was reduced to 1250 °C, and the platinum-rhodium crucible filled with the glass melt was taken out from the furnace, moved to a higher position and tilted slightly to allow the glass melt to flow out naturally. The flow of the glass melt rapidly cooled in ambient air, forming continuous precursor glass fibers with a diameter of approximately 150 μm. Then, the fiber segment was heated using a CO_2_ laser to induce localized softening of the fiber. Under gravitational force, the softened region stretched into a tapered section with reduced diameter. Precise control over the taper waist diameter was achieved by modulating the laser power and the fiber-to-focus distance. The tapered fiber was then cleaved to the desired lengths using a focused CO_2_ laser beam. Subsequently, instantaneous CO_2_ laser irradiation was used to melt the fiber tip, and a microsphere was produced due to the surface tension of the glass melt. The diameter of the as-prepared microsphere was precisely controlled via focus distance manipulation using 3-axis adjustment stage during the laser-assisted fabrication process. Finally, the microcavities were placed in a high-purity quartz petri dish and crystallized by heating in a muffle furnace at different temperatures for 1.5 hours to precipitate the BTG microcrystals.

### Characterizations

Differential scanning calorimetry analysis was performed with a thermal analyzer (STA449C/3/MFC/G, NETZSCH) with a heating rate of 10 K min^−1^ under ambient atmosphere. The refractive indexes of GC samples were measured with a prism coupler (Model 2010, Metricon). The crystallization process was identified with an X-ray diffractometer (PANalytical, Netherlands) with Cu/K*α* radiation. Raman spectra were obtained from a Raman spectrometer (inVia, Renishaw) under the excitation of a 785 nm laser. A scanning electron microscope (JSM-7900F, JEOL, Japan) was employed to determine the morphology of microspheres. The microstructure and element distribution of the microcrystals were measured using a high-resolution transmission electron microscope (JEM-2100F, JEOL, Japan) equipped with an energy dispersive spectrometer. Transmission spectra of the bulk samples were obtained from a Perkin-Elmer Lambda 900 UV/VIS/NIR spectrophotometer (MA, Waltham). Upconversion fluorescence spectra were recorded by a fiber optic spectrometer (QE65000, Ocean Optics). The fluorescence decay curves were recorded by the digital oscilloscope (TDS 3012c, Tektronix) under the excitation of a pulsed 980 nm laser. A tunable optical parametric amplification femtosecond laser (ORPHEUS-HP, Light Conversion) with a repetition frequency of 50 kHz and a pulse width of 140 fs was employed as the SHG excitation light source. The UC laser and SHG signal were recorded by a high-resolution spectrometer (monochromator: Omni-λ3024i, Zolix & CCD: Newton 920, ANDOR). The SHG properties of the microcavities are collected using the free-space pumping scheme. The excited area is near the edge of the microcavity, and the detection area is on the other side, normally showing a bright SHG signal. The SHG conversion efficiency was defined as the ratio of the output signal power collected from the tapered fiber to the input pump power of the femtosecond laser transmitted through the objective lens. The pump beam spot diameter is calculated to be 4.86 μm through the formula *d* = 4M^2^*λf*/(π*ω*), where M^2^ value of the femtosecond laser is 1.04, input laser wavelength *λ* = 1.1 μm, focal length *f* of the objective is 10 mm, and diameter *ω* of the beam input to the objective is 3 mm.

## Supplementary information

Supporting information

## Data Availability

All data needed to evaluate the findings of this study are available within the Article and Supplementary Information. Source Data files are also available upon request.
