# Supplementary material for: A monolithic microcavity laser with simultaneous upconversion and frequency-doubled lasing via crystal-in-glass engineering
Source: Light Sci Appl. 2026 Jan 26;15:86. doi: 10.1038/s41377-025-02162-9 (PMC12832608; doi:10.1038/s41377-025-02162-9)
Supplement: Supplementary file 1 — Supporting information [file 41377_2025_2162_MOESM1_ESM.docx]

Supporting Information for

**A monolithic microcavity laser with simultaneous upconversion and frequency-doubled lasing via crystal-in-glass engineering**

Shengda Ye^1^, Jianhao Chen^1^, Jiayue He^1^, Weiwei Chen^2^, Xiongjian Huang^1, 3^, Xiaofeng Liu^4^, Jianrong Qiu^5^, Zhongmin Yang^3^, and Guoping Dong^1^*

^1^State Key Laboratory of Luminescent Materials and Devices, School of Materials Science and Engineering, South China University of Technology, Guangzhou 510640, China.

^2^School of Optoelectronic Engineering, Guangdong Polytechnic Normal University, Guangzhou 510665, China.

^3^School of Physics and Optoelectronics, South China University of Technology, Guangzhou 510640, China.

^4^School of Materials Science and Engineering, Zhejiang University, Hangzhou 310027, China.

^5^State Key Laboratory of Modern Optical Instrumentation, College of Optical Science and Engineering, Zhejiang University, Hangzhou 310027, China.

Correspondence: Guoping Dong (dgp@scut.edu.cn)

**S1 The crystallinity analysis of different GC samples.**

**
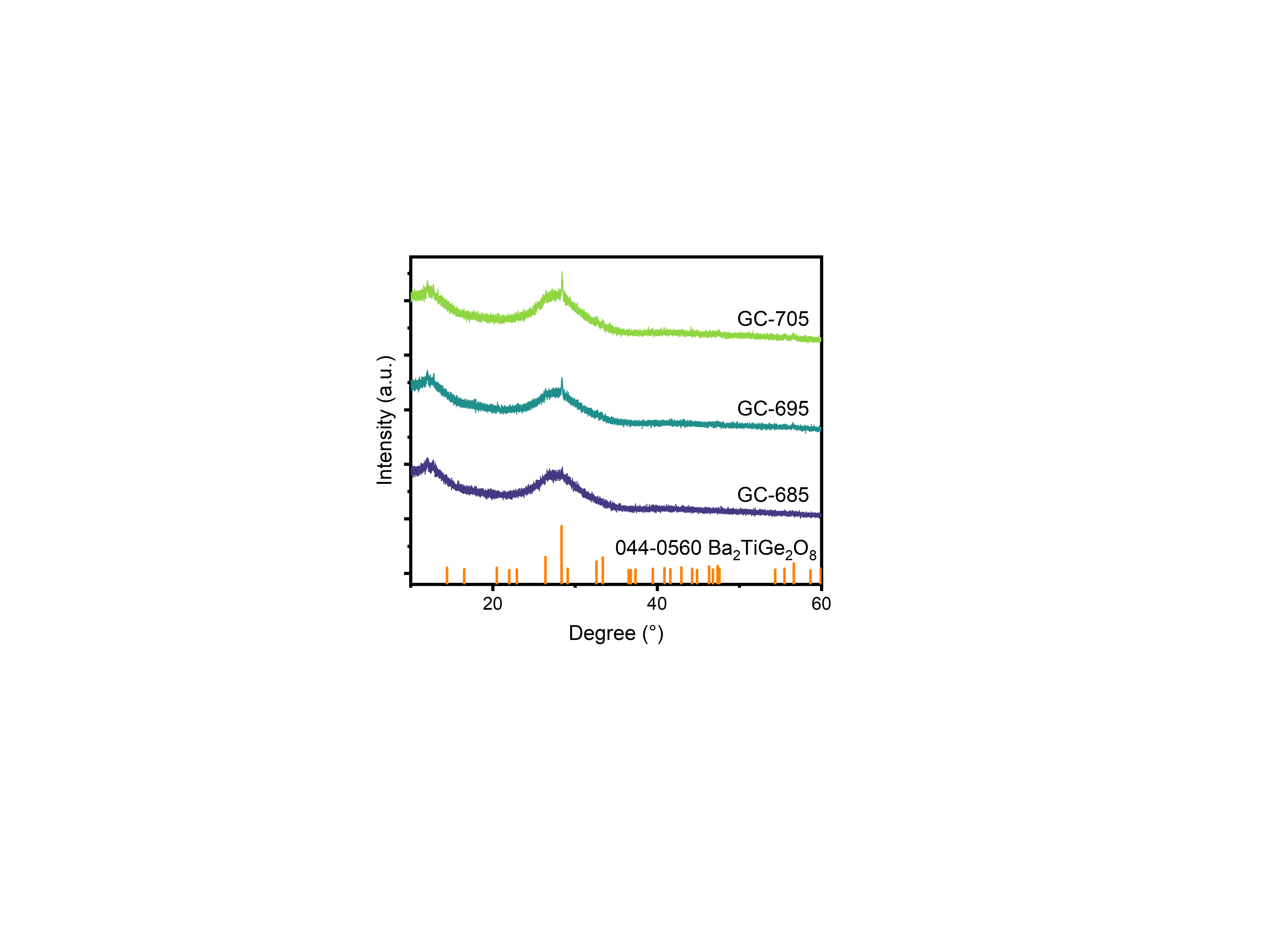
**

**Fig. S1** XRD patterns of GC-685, GC-695, and GC-705 samples.

**Table S1** Crystallinity of GC-685, GC-695, and GC-705 samples.

| Sample | Heat treatment | Crystallinity (%) |
| --- | --- | --- |
| GC-685 | 685 °C/1.5h | 0.55 |
| GC-695 | 695 °C/1.5h | 2.66 |
| GC-705 | 705 °C/1.5h | 4.58 |

**S2 Transmittance and absorption characterization**

As shown in Fig. S2a, as the heat treatment temperature increases, the transmittance of the sample gradually decreases. Among them, the transmittance of samples GC-685 and GC-695 remained around 80% in the visible wavelength range. When the heat treatment temperature rose to 705 °C, the transmittance of the sample dropped to around 70% due to the more severe scattering lose caused by the high degree precipitation of Ba_2_TiGe_2_O_8_ crystals.

The characteristic absorption peaks of the doped rare earth ions shown in the transmittance spectra including 378 nm (^4^I_15/2_ → ^4^G_11/2_), 408 nm (^4^I_15/2_ → ^2^H_9/2_), 452 nm (^4^I_15/2_ → ^4^F_5/2_), 489 nm (^4^I_15/2_ → ^4^F_7/2_), 521 nm (^4^I_15/2_ → ^2^H_11/2_), 547 nm (^4^I_15/2_ → ^4^S_3/2_), 654 nm (^4^I_15/2_ → ^4^F_9/2_), and 800 nm (^4^I_15/2_ → ^4^I_9/2_) peaks, which are characteristic absorption bands of Er^3+^. Additionally, the absorption peak at 978 nm corresponds to the transitions ^4^I_15/2_ → ^4^I_11/2_ of Er^3+^ and ^4^F_7/2_ → ^4^F_5/2_ of Yb^3+^.


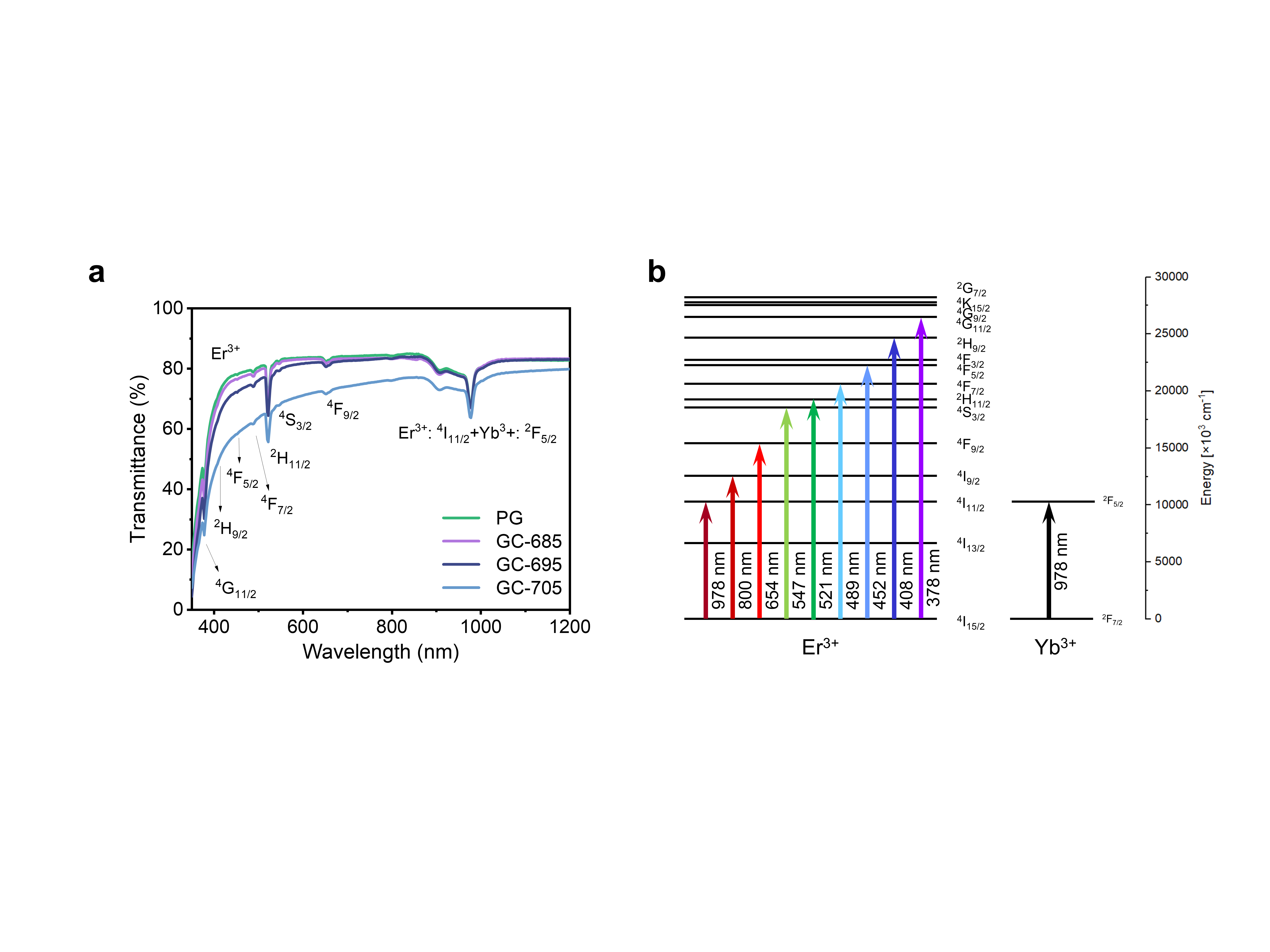


**Fig. S2 a** Transmittance spectra of PG, GC-685, GC-695, and GC-705 samples. **b** Energy level diagrams of Er^3+^ and Yb^3+^ with the characteristic absorption transition.

**S3 UC emission properties**

The UC emission intensity of PG and GCs are shown in Fig. S3. The heat treatment does not significantly affect the UC emission intensity. This is attributed to the similar stoichiometry between the glass matrix and the precipitated Ba₂TiGe₂O₈ crystals that the crystallization would not cause severe element segregation, which results in a nearly identical coordination environment for the rare-earth ions.


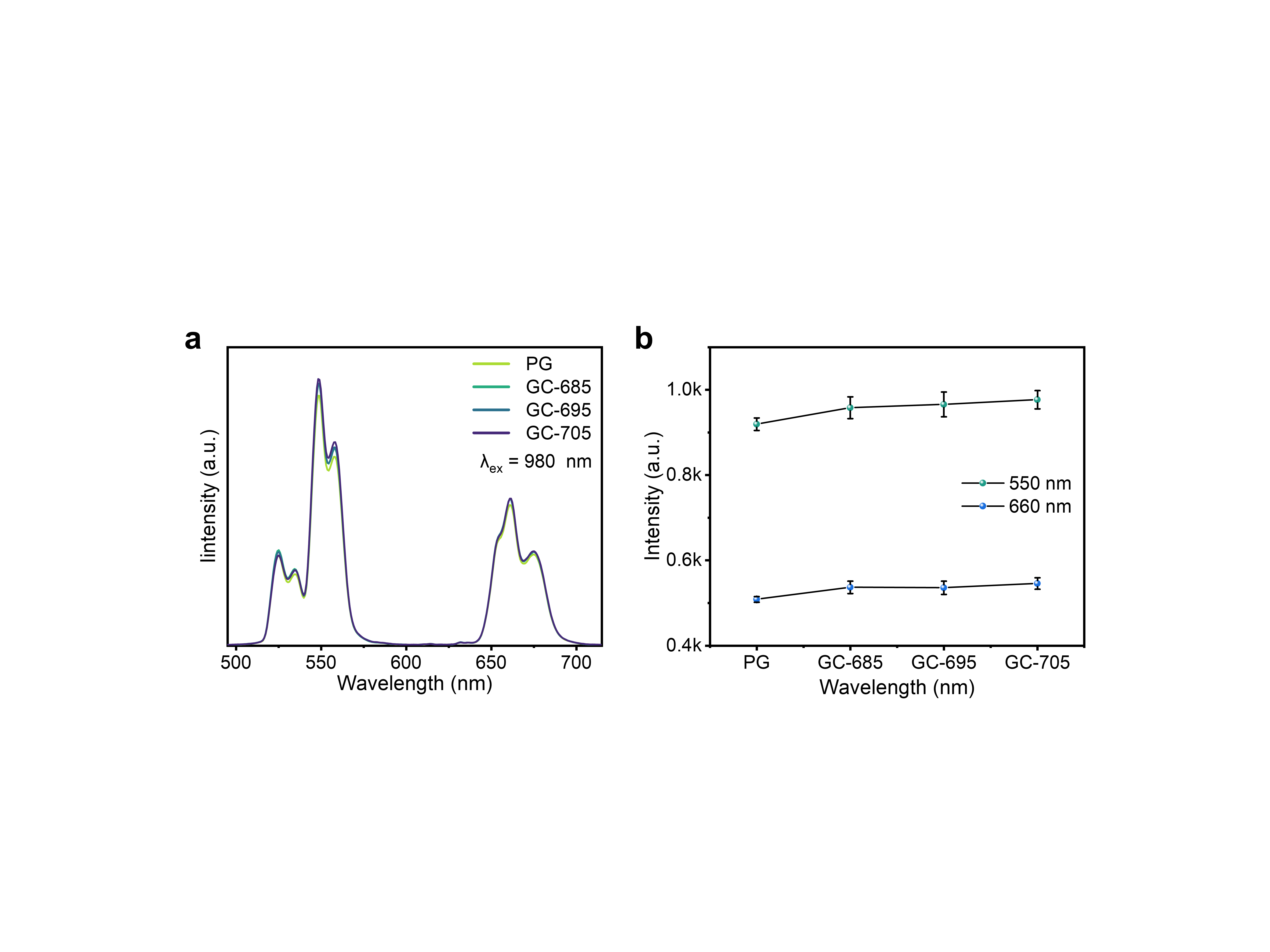


**Fig. S3** **a** UC emission spectra, **b** emission peak intensity (550 nm and 660 nm) of PG, GC-685, GC-695, and GC-705 samples with error bar showing the standard deviation.

The relationship between UC fluorescence intensity *I* and pump power *P* can be expressed as *I* ∝ *P^n^*, where *n* reflects the number of photons involved in the excitation process^1,2^. As shown in Fig. S4b and S4c, the *n* values for the 550 nm and 660 nm emissions were obtained through linear fitting of the power dependence of intensity relations plotted in the double logarithmic scale. For the BTG: 0.5YbF_3_/0.1ErF_3_ system, the *n* values for the 550 nm and 660 nm emissions in the PG are 2.01 and 1.85, respectively, while for the GC-695 sample, they are 2.03 and 1.91, respectively. These results agree with process of multi-level energy transfer from Yb^3+^ to Er^3+^ (Fig. S4a).


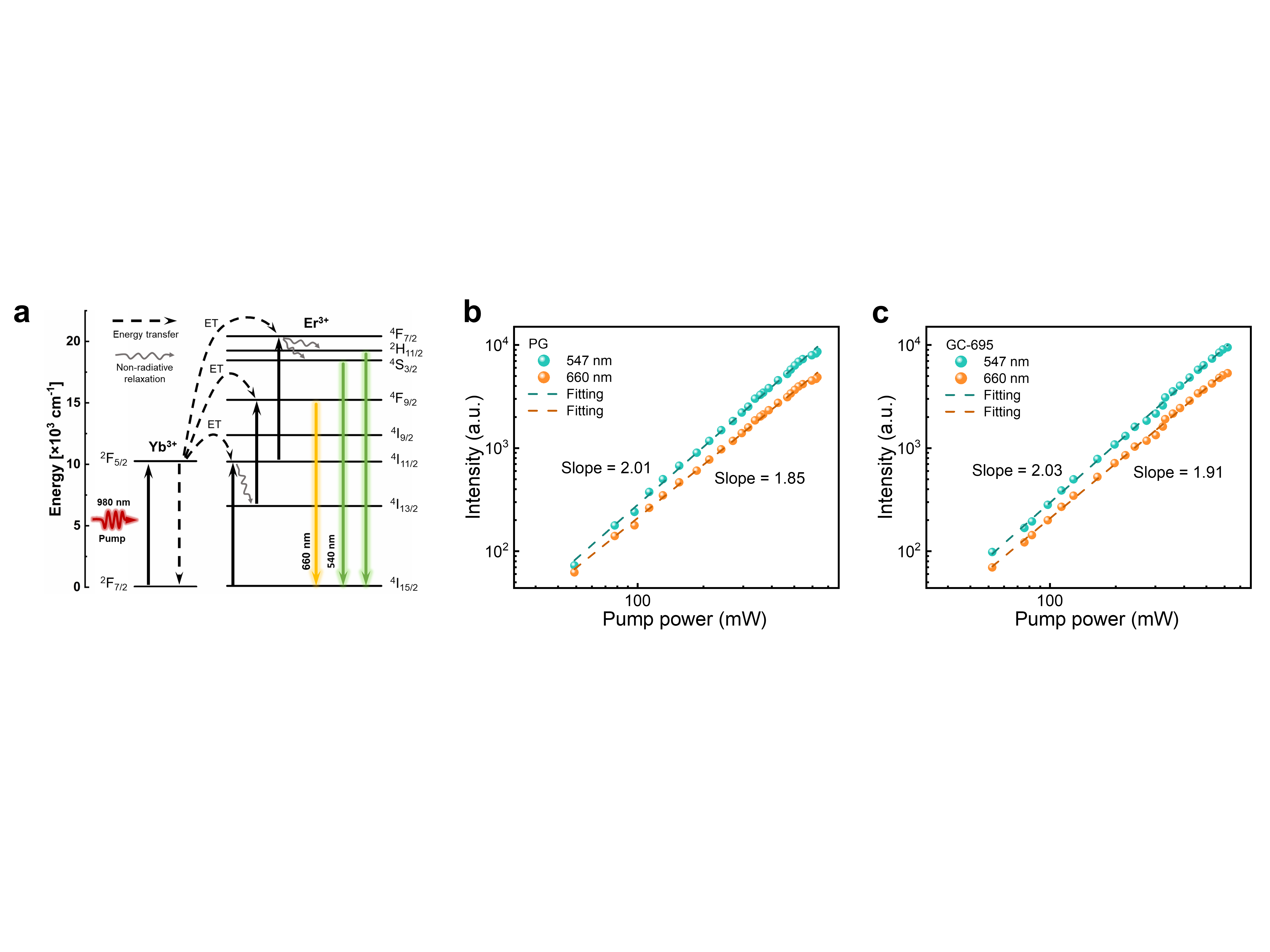


**Fig. S4** **a** Upconversion energy level diagrams of Er^3+^ and Yb^3+^. Excitation power dependence of UC fluorescence intensity plotted in double logarithmic scale for Er^3+^ and Yb^3+^ co-doped **b** PG and **c** GC-695 samples.

**S4 SHG intensity of PG, GC-685, GC-695, and GC-705 samples.**

**
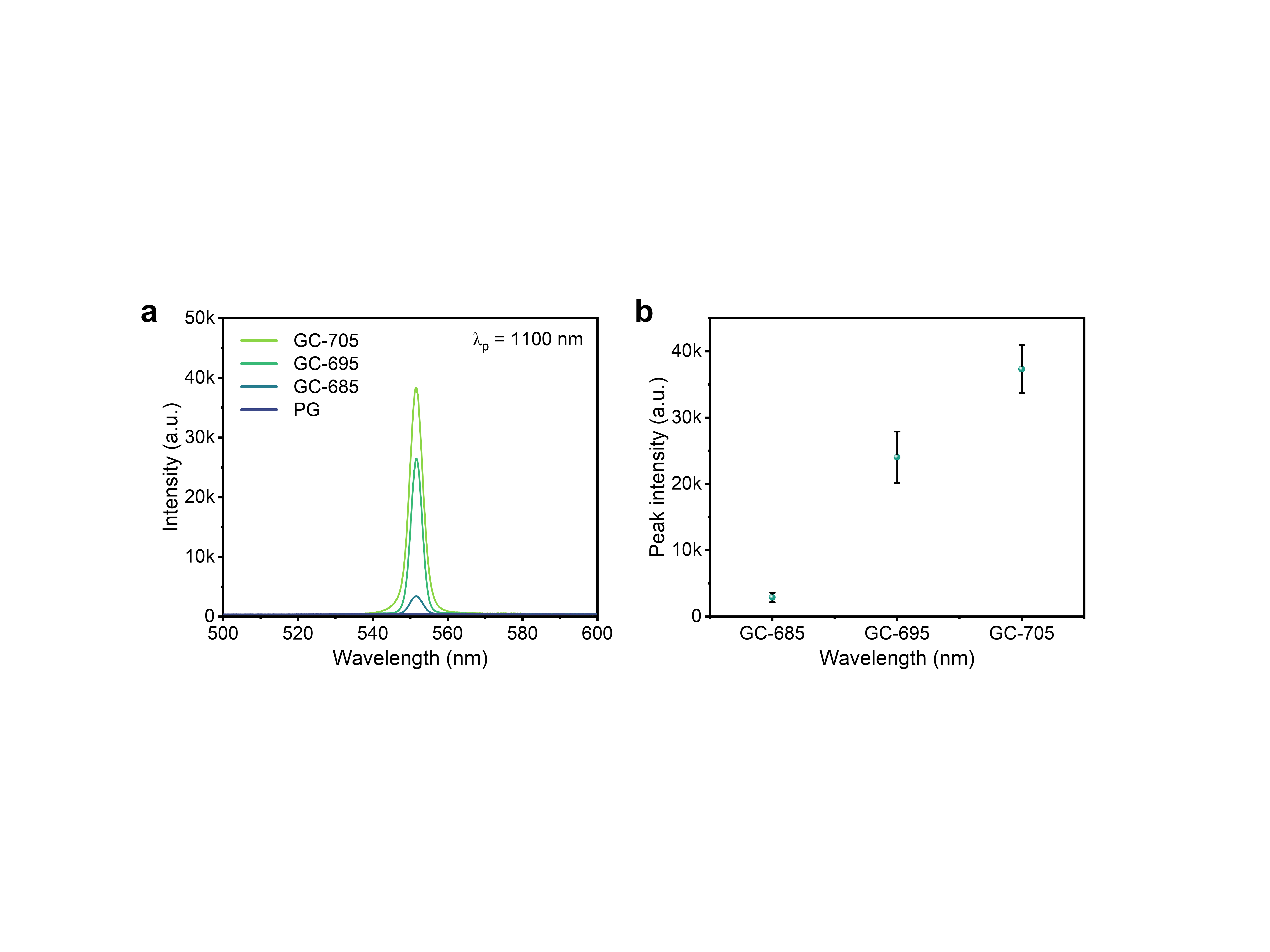
**

**Fig. S5** **a** SHG intensity spectra, **b** peak intensity of PG, GC-685, GC-695, and GC-705 samples with error bar showing the standard deviation. Femtosecond laser pumping wavelength λ_p_ is 1100 nm.

**S5 The micrographs of the precursor and other samples with different heat-treated temperature**


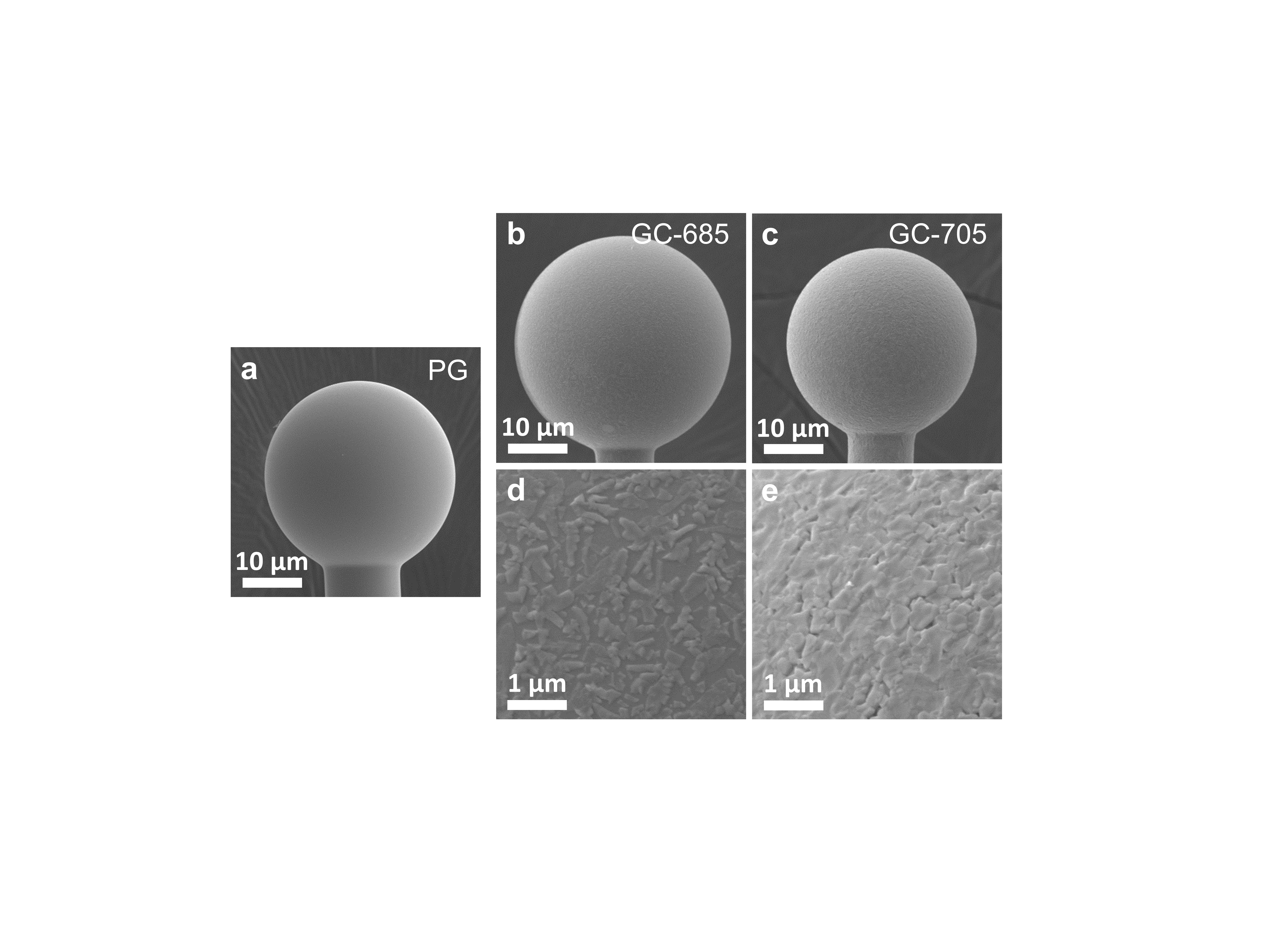


**Fig. S6** SEM images of **a** PG, **b** GC-685, and **c** GC-705 microcavities. **d**, **e** Surface morphologies of GC-685 and GC-705 samples.

**S6 Q factor of PG and GC samples.**


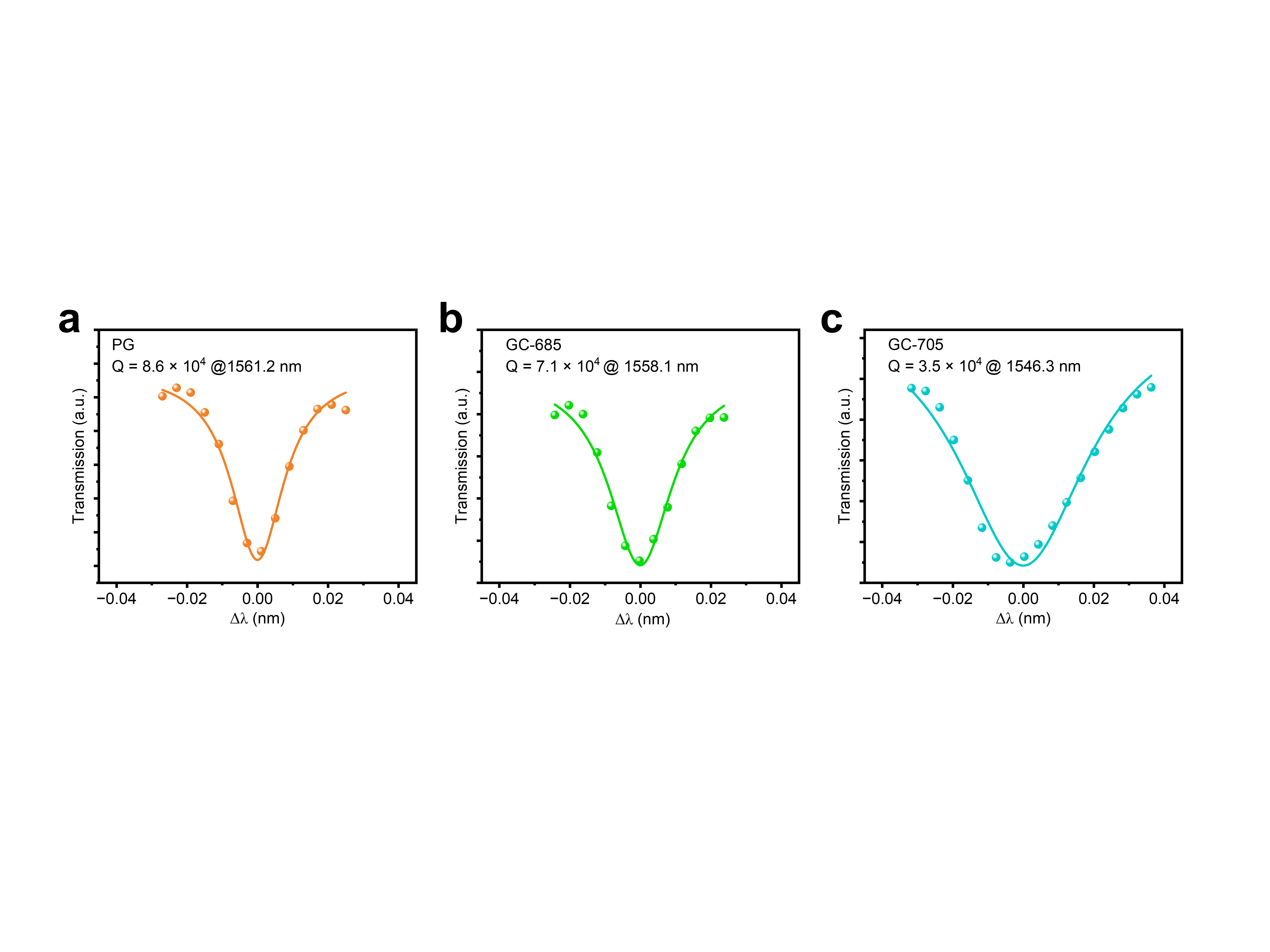


**Fig. S7** Q factor of **a** PG, **b** GC-685, and **c** GC-705 samples.

**S7 Refractive indices and free space range (FSR) calculation**

**Table** **S2** Refractive index 𝑛 of the GC-695 samples.

| Wavelength  [μm] | GC-695 | GC-695-1 | GC-695-2 | | GC-695-3 | | GC-695-4 | Standard deviation |
| --- | --- | --- | --- | --- | --- | --- | --- | --- |
| 0.6328 | 1.83461 | 1.83521 | | 1.83501 | | 1.8347 | 1.83430 | 3.54443 × 10^-4^ |
| 1.309 | 1.81293 | 1.81281 | | 1.81253 | | 1.81255 | 1.81212 | 3.12282 × 10^-4^ |
| 1.533 | 1.81222 | 1.81249 | | 1.81248 | | 1.81224 | 1.81165 | 3.41218 × 10^-4^ |

The refractive index 𝑛 of the polished GC-695 sample at different wavelength 𝜆 was obtained by using Cauchy dispersion formula fitting according to following Equation:

$n=1.81113+\frac{0.00116}{\lambda^{2}}+\frac{0.0033}{\lambda^{4}}$ (1)

**Table** **S3** Fitting coefficients of the Cauchy dispersion formula.

| Fitting coefficients |  | Standard error |
| --- | --- | --- |
| a | 1.81113 | - |
| b | 0.00116 | 4.29488 × 10^-7^ |
| c | 0.0033 | 1.78689 × 10^-7^ |


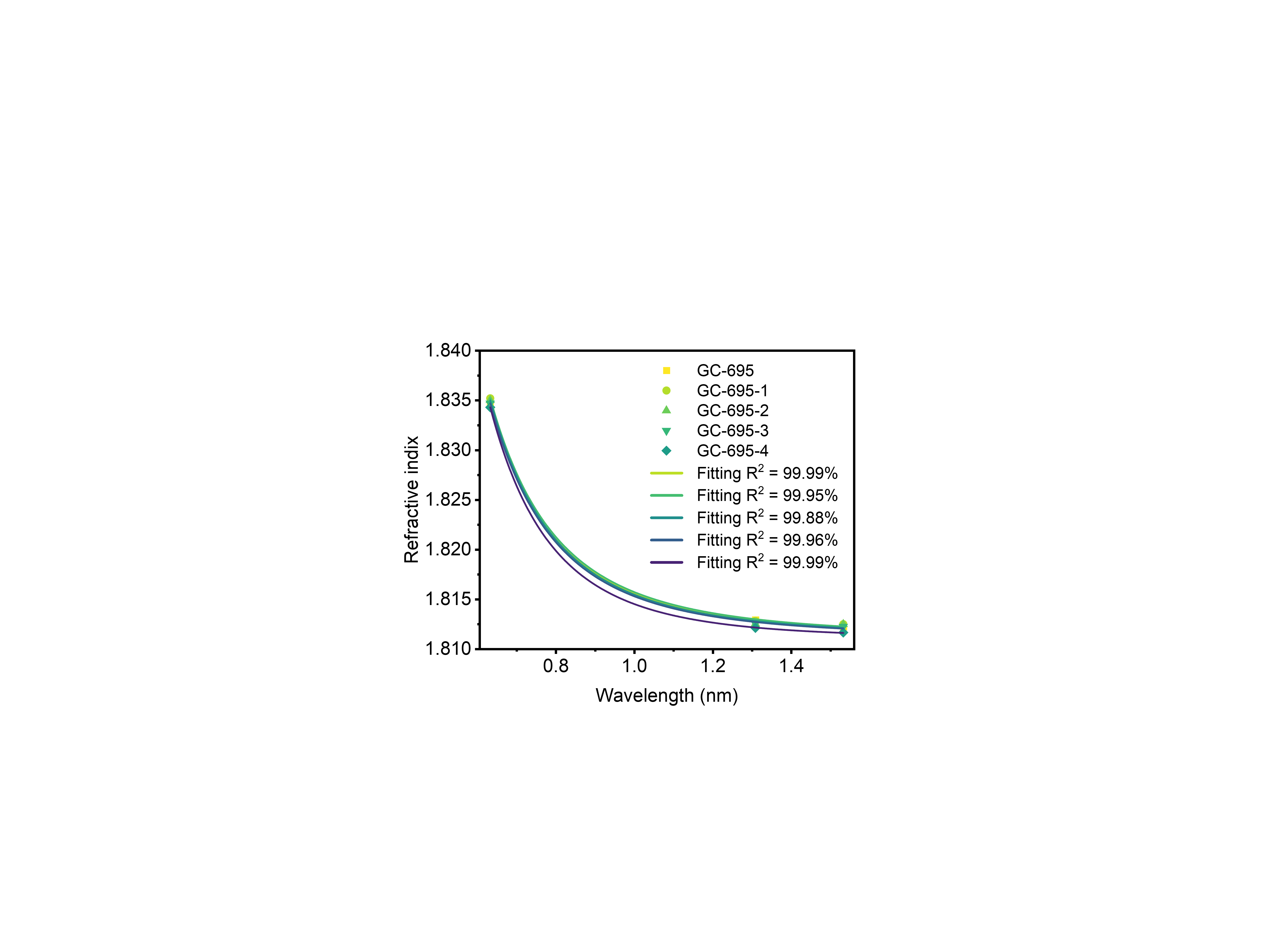


**Fig. S8** Refractive index of the GC-695 samples. The fitting curves and correlation coefficient R^2^ are shown.

The refractive indices for the Er^3+^/Yb^3+^ co-doped BTG GC-695 were calculated as 1.87321 at 490 nm, 1.85103 at 550 nm, 1.83118 at 660 nm, and 1.81592 at 980 nm. To further analyze the microcavity performance, the FSR was calculated by^3^:

$FSR=\frac{\lambda_{0}^{2}}{n\pi D}$ (2)

where *λ_0_* is the central wavelength of the WGM, *n* is the refractive index of the material, and *D* is the microcavity diameter. The calculated *n* values at 550 nm and 660 nm are used. The calculated FSR at the 550 nm and 660 nm bands are 1.73 nm and 2.52 nm for the 30 μm microcavity, respectively, and 1.16 nm and 1.68 nm for the 45 μm microcavity, respectively.

**Table S4** Comparison of UC microlasers based on microspheres with different materials.

| Materials | Laser band (nm) | Diameter (μm) | Q factor | Threshold |
| --- | --- | --- | --- | --- |
| Er doped ZBLAN microsphere^4^ | 539 | 120 | 10^6^ | 30 μW |
| Er doped ZBLAN microsphere^5^ | 550 | 60 | 10^3^ | 3 μW |
| NaGdF_4_:Er/Yb@NaGdF_4_ nanopaticles surface-coated PS microspheres^6^ | 557  665 | 5 | 10^3^ | 40 W cm^-2^ |
| Er-doped silica glass microspheres^7^ | 380  410 | 56 | 1.2 × 10^8^ | 600 μW  176 μW |
| Tm-Er-Yb doped PMMA surface-coated SiO2 microspheres^8^ | 480  550  660 | 53 | 1.6 × 10^7^ @ 34 nm  2.2 × 10^7^ @ 766 nm  1.8 × 10^7^ @ 1621 nm | 300 μW  500 μW  90 μW |
| Er-Yb doped PMMA surface-coated SiO_2_ microspheres^9^ | 450  560  660  800 | 57 | 1.2 × 10^8^ | 2.7 μW  19 μW  0.18 μW  1.7 μW |
| Er/Yb co-doped GCs microspheres^3^ | 550  660 | 15 | 0.33 × 10^5^(Ho/Yb co-doped) | 15.8 μW  13.4 μW |
| Er/Yb co-doped GCs microspheres^10^ | 545  650 | 30 | 1.85 × 10^5^ | 150 μW  157 μW |
| Er/Yb co-doped GCs microspheres  (this work) | 547  660 | 30 | 0.57 × 10^5^ | 13.31 μW  12.97 μW |

PS: polystyrene PMMA: polymethyl methacrylate

**S8** **The pump polarization dependence measurement of UC laser**

The UC lasing was excited by a polarized 980 nm laser coupled into the microsphere via free-space pumping at the edge of the microsphere. The output signal was collected using a tapered fiber. Across different polarization angles of the pump, the UC lasing intensity remained nearly constant, confirming its polarization-independent character.


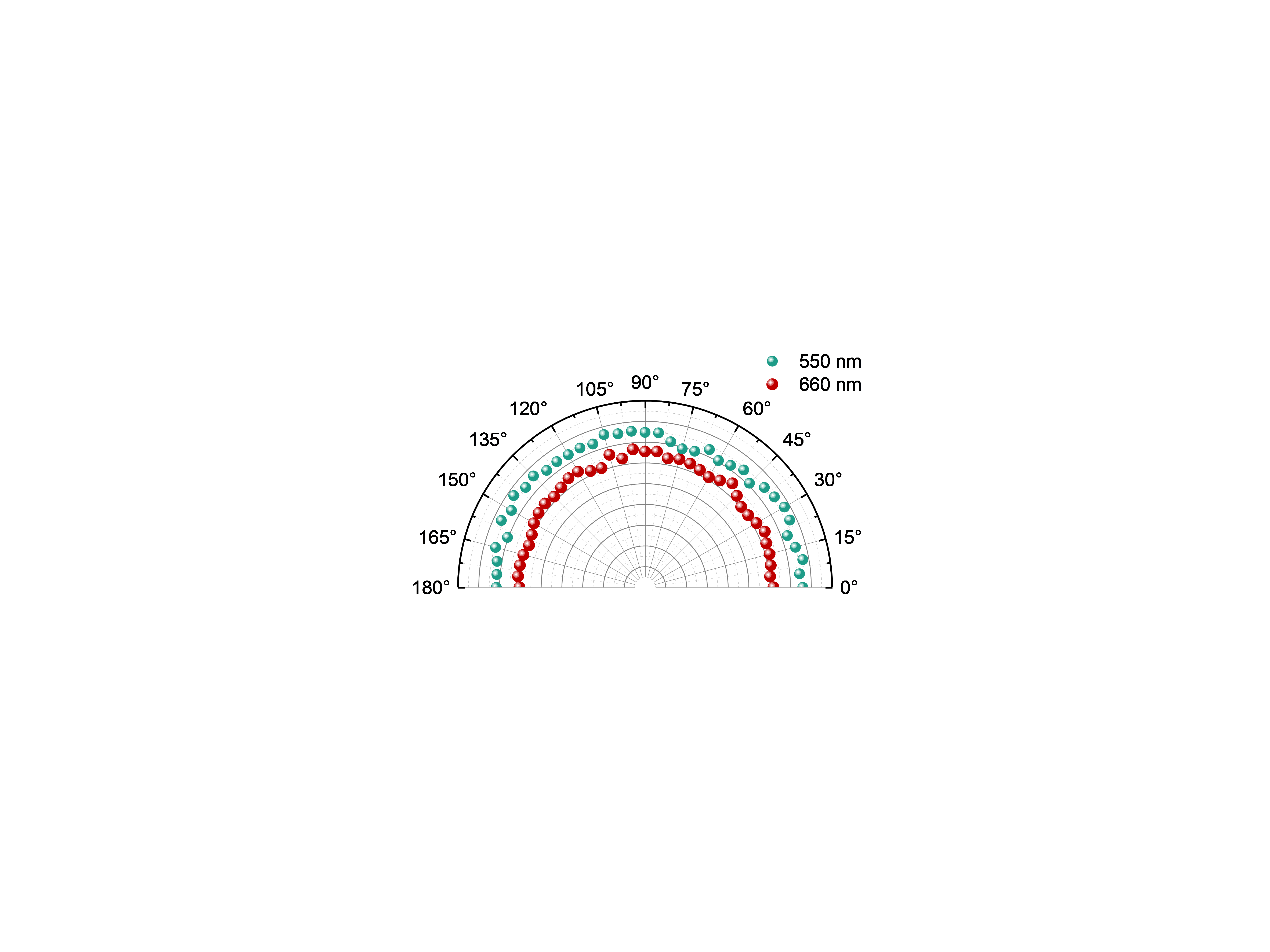


**Fig. S9** Polar plot of the UC lasing intensity for the GC-695 microcavity as a function of 980 nm excitation polarization.

**S9 UC laser output of Er^3+^/Yb^3+^ co-doped BTG GC microcavity with a diameter of 45 μm**


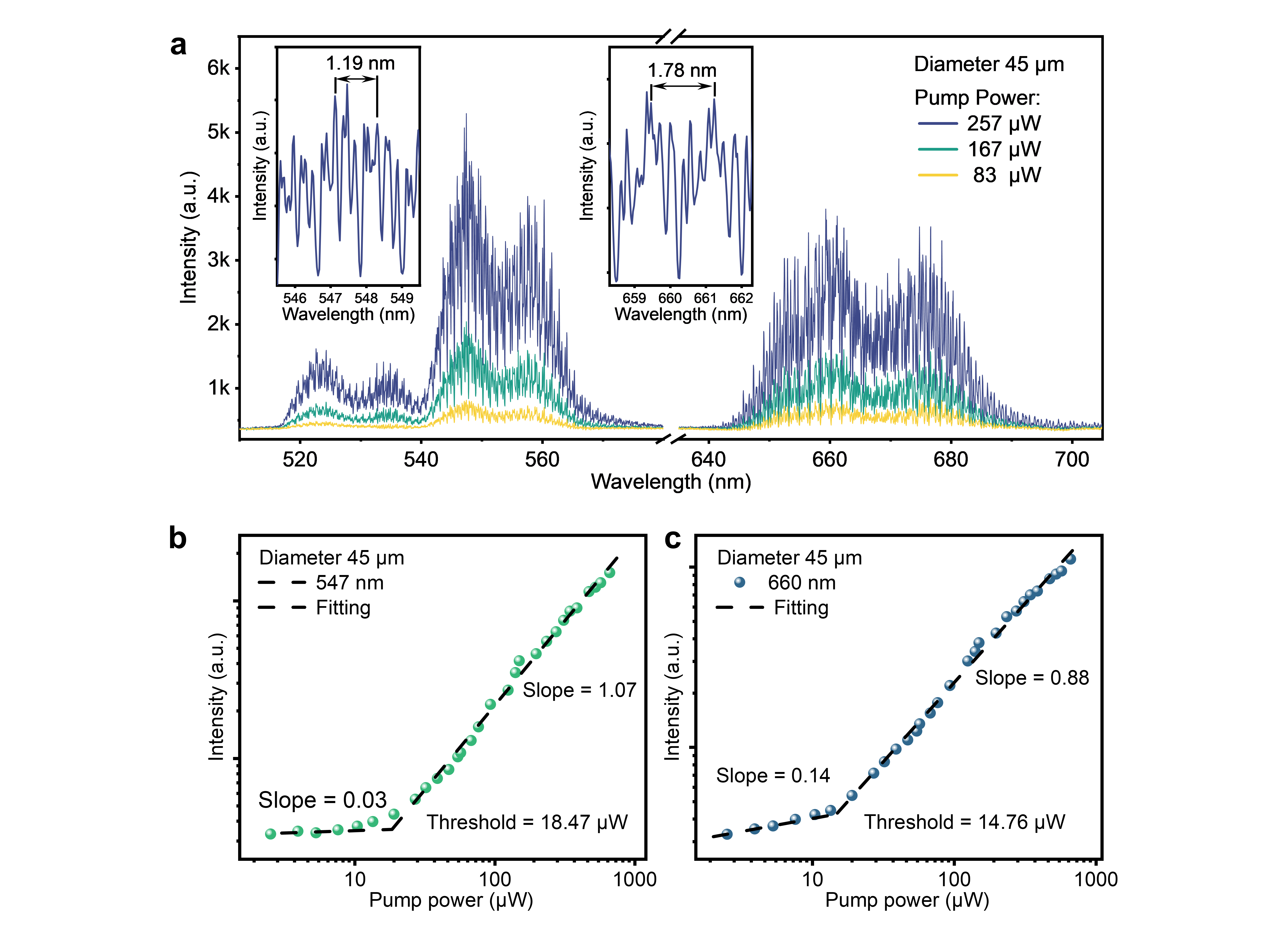


**Fig. S10** **UC lasing based on the GC-695 microcavity with a diameter of 45** **μm.** **a** UC lasing spectra of the GC-695 microcavity at different pumping powers. Insets show the zoom-in laser spectra. **b**, **c** Pump power dependence of lasing intensity of the GC-695 microcavity at 550 nm and 660 nm, respectively.

**S10 Influence of** **femtosecond laser on UC Lasing**

Fig. S11 shows the power dependence curves at 547 nm of three microspheres before and after adding the femtosecond laser pump. The lasing thresholds remained consistent after adding the femtosecond laser pump, with no systematic variation. Fig. S12 shows the lasing spectra before and after adding the femtosecond laser pump. As can be seem, the mode structure and intensity remained stable, indicating the robust dual-mode lasing operation under femtosecond laser pumping.


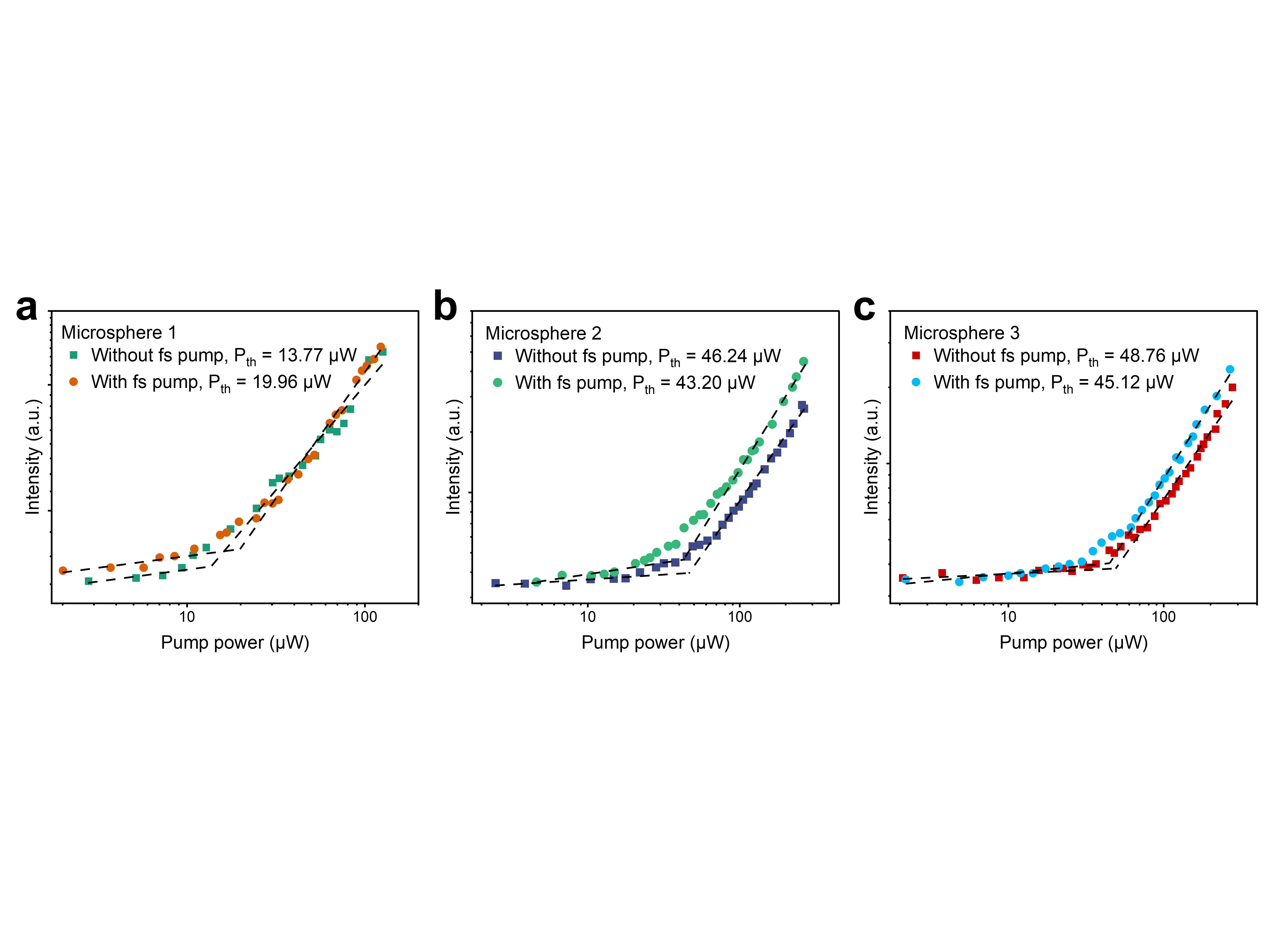


**Fig. S11** Power dependence curves at 547 nm with and without femtosecond laser pump.


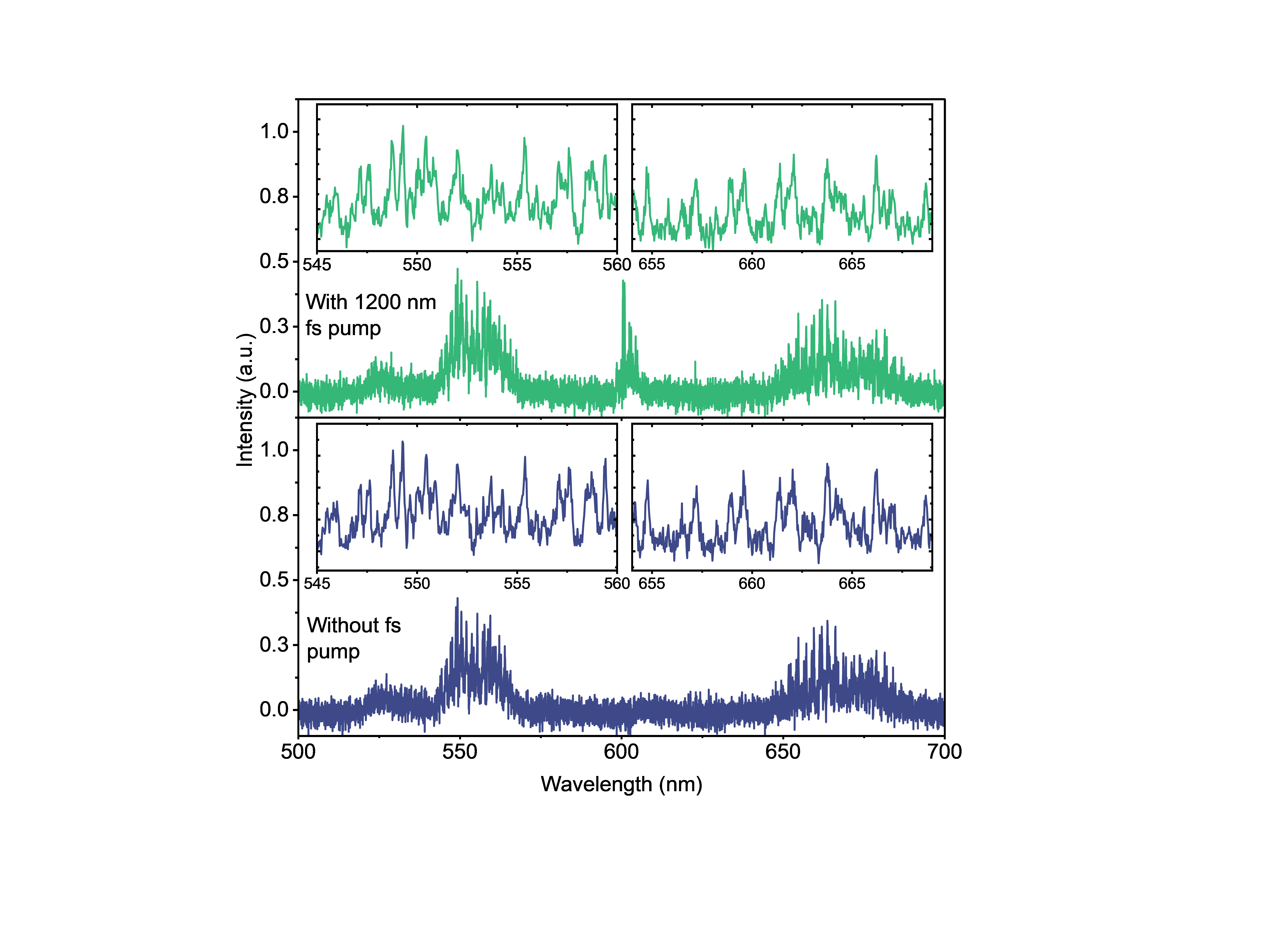


**Fig. S12** Lasing spectra with and without femtosecond laser pump.

**S11 UC lasing and SHG dual-mode output spectra**

By enhancing the UC lasing intensity, CIE coordinates with different tunable range can be obtained. Figure S14 shows the CIE coordinates of the dual-mode output with different femtosecond pump wavelength, from which more precise tunable range in the inner dashed circle 1 can be observed. The dashed circle 2 refer to the tunable range with larger SHG and UC lasing intensity ratio whose spectra are shown in Fig. 6.


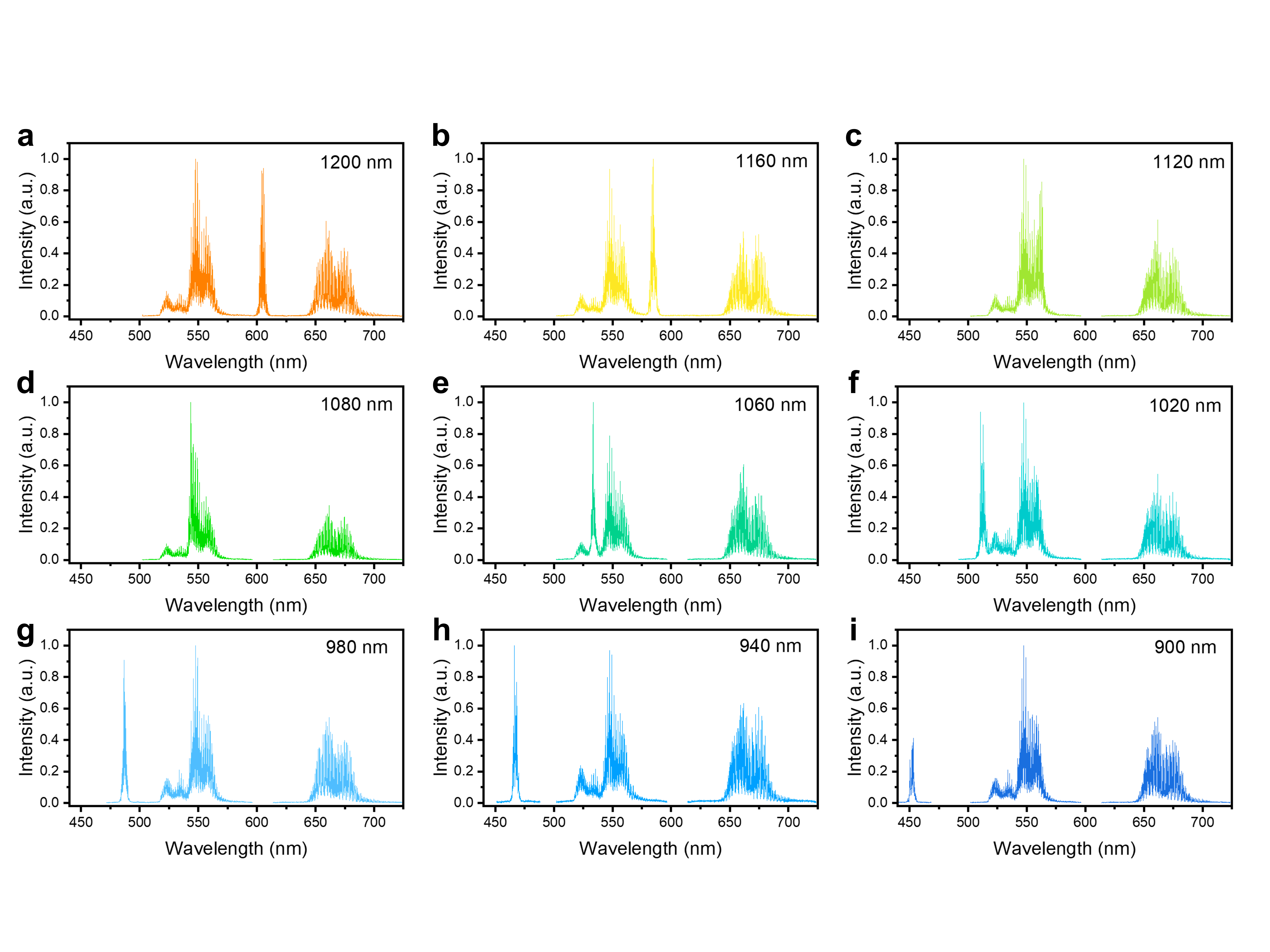


**Fig. S13** Dual-mode lasing output spectra of the GC microsphere with different femtosecond pulse pump wavelength of **a** 1200 nm, **b** 1160 nm, **c** 1120 nm, **d** 1080 nm, **e** 1060 nm, **f** 1020 nm, **g** 980 nm, **h** 940 nm, and **i** 900 nm.


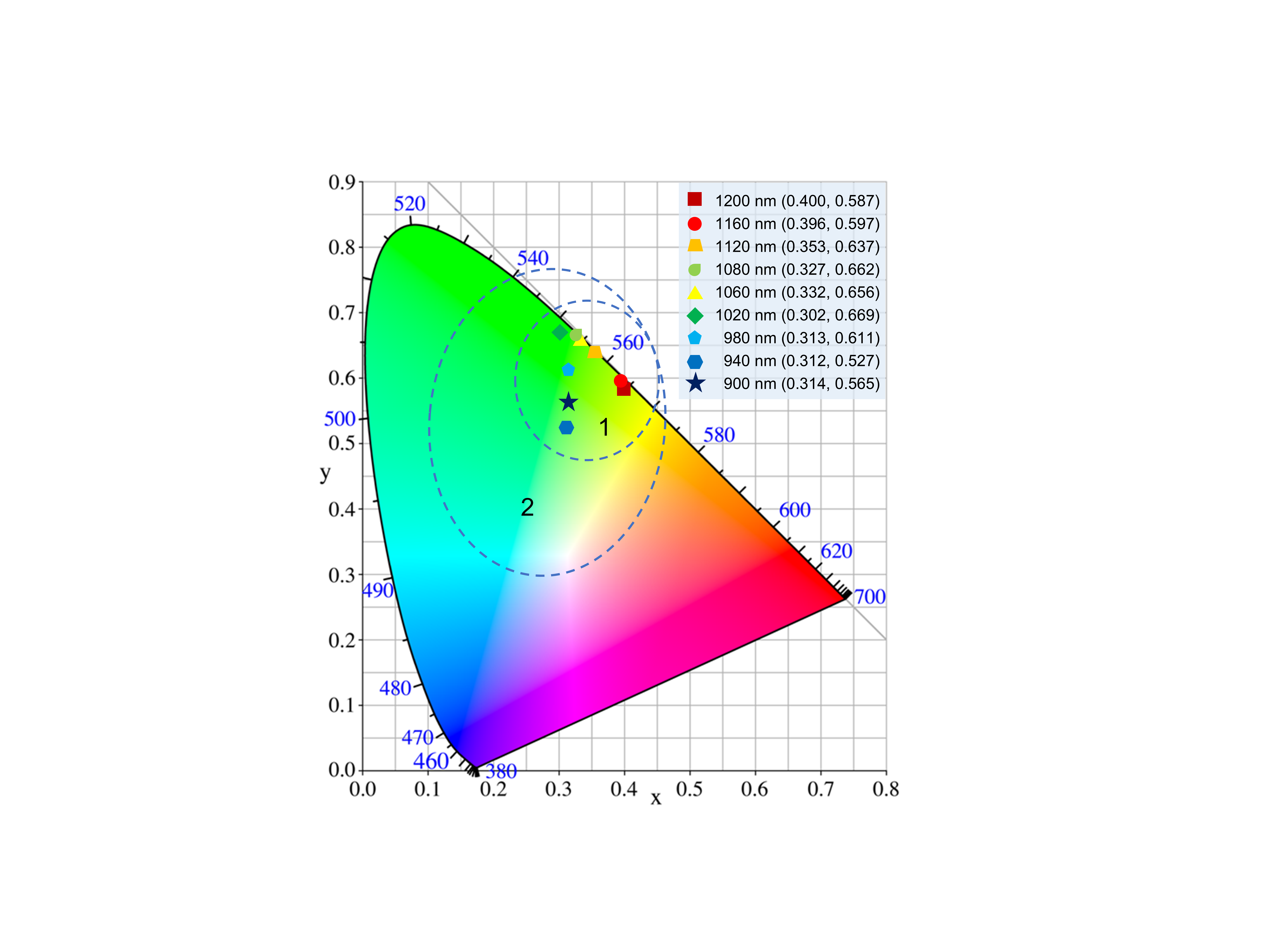


**Fig. S14** Tunable CIE coordinates of the dual-mode microcavity laser calculated from the spectra shown in Fig. S13.

**References**

1. Wang, D. Y. et al. Efficient down- and up-conversion luminescence in Er^3+^-Yb^3+^ co-doped Y_7_O_6_F_9_ for photovoltaics. *ACS Applied Energy Materials* **1**, 447-454 (2018).

2. Zhang, Y. et al. Dual-wavelength enhanced upconversion luminescence properties of Li^+^-doped NaYF_4_:Er,Yb glass-ceramic for all-optical logic operations. *Dalton Transactions* **52**, 2948-2955 (2023).

3. Chen, J. H. et al. Boosted continuous-wave upconversion microlaser based on Yb^3+^/Ho^3+^ co-doped oxyfluoride glass-ceramics with NaYF_4_ nanocrystals. *Laser & Photonics Reviews* **19**, 2402218 (2025).

4. Von Klitzing, W. et al. Very low threshold lasing in Er^3+^ doped ZBLAN microsphere. *Electronics Letters* **35**, 1745-1746 (1999).

5. Wu, Y. Q., Ward, J. M. & Nic Chormaic, S. Ultralow threshold green lasing and optical bistability in ZBNA (ZrF_4_–BaF_2_–NaF–AlF_3_) microspheres. *Journal of Applied Physics* **107**, 033103 (2010).

6. Yang, X. F. et al. Lanthanide upconverted microlasing: Microlasing spanning full visible spectrum to near-infrared under low power, CW pumping. *Small* **17**, 2103140 (2021).

7. Jiang, B. et al. Four- and five-photon upconversion lasing from rare earth elements under continuous-wave pump and room temperature. *Nanophotonics* **11**, 4315-4322 (2022).

8. Jiang, B. et al. Room-temperature continuous-wave upconversion white microlaser using a rare-earth-doped microcavity. *ACS Photonics* **9**, 2956-2962 (2022).

9. Jiang, B. et al. Simultaneous ultraviolet, visible, and near-infrared continuous-wave lasing in a rare-earth-doped microcavity. *Advanced Photonics* **4**, 046003 (2022).

10. Gao, Z. G. et al. Robust low threshold full-color upconversion lasing in rare-earth activated nanocrystal-in-glass microcavity. *Light: Science & Applications* **14**, 14 (2025).
